# Exploring the gut microbiome and metabolomic interactions of antimetabolite drugs to optimize therapy

**DOI:** 10.1080/19490976.2026.2638009

**Published:** 2026-02-27

**Authors:** Jingyang Chen, Yanan Wang, Lei Xu, Xiaona Li, Libo Zhao

**Affiliations:** a Department of Pharmacy, Peking University Third Hospital, Beijing, People's Republic of China; b Therapeutic Drug Monitoring and Clinical Toxicology Center of Peking University, Beijing, People's Republic of China

**Keywords:** Antimetabolite drugs, gut microbiota, efficacy, toxicity, biomarker, intervention

## Abstract

Antimetabolite drugs are cornerstones in treating various cancers and autoimmune diseases; however, their clinical utility is often hampered by systemic toxicity caused by drug-induced gut microbiota dysbiosis. Predicting patient responses remains a significant challenge. Several studies have highlighted the influence of gut microbiota on antimetabolite treatment outcomes, revealing complex bidirectional interactions between the drugs and microbial communities. This review synthesizes the effects of common antimetabolites (including 5-fluorouracil, methotrexate, gemcitabine, capecitabine, 6-mercaptopurine, and thioguanine) on gut microbial communities and outlines a framework (pharmacokinetics, endogenous metabolite production, immune modulation, and apoptotic pathway modulation) for assessing chemotherapy-microbiota interactions. Additionally, potential microbial biomarkers for predicting treatment responses and strategies for manipulating the gut microbiota to enhance therapeutic efficacy are discussed. Therefore, advances in methodologies such as metagenomics and real-time microbial monitoring will be essential for unraveling these interactions and promoting the precise application of antimetabolite drugs.

## 1. Introduction

Antimetabolite drugs are widely used chemotherapeutic agents that mimic the molecular structures of substances involved in nucleic acid synthesis, inhibiting cell division and proliferation. Antimetabolite drugs can be classified into five categories, based on the different target enzymes they inhibit: ^1^ thymidylate synthase (TS) inhibitors, such as 5-fluorouracil (5-FU);^2^ dihydrofolate reductase (DHFR) inhibitors, such as methotrexate (MTX);^3^ purine nucleotide interconversion inhibitors, such as mercaptopurine (6-MP);^4^ ribonucleotide reductase inhibitors, such as hydroxyurea; and ^5^ deoxyribonucleic acid (DNA) polymerase inhibitors, such as cytarabine. Owing to their broad cytotoxic activity, antimetabolite drugs are used to treat various malignancies (including colorectal cancer [CRC], breast cancer, leukemia, choriocarcinoma, and osteosarcoma) and inflammatory conditions (such as inflammatory bowel disease [IBD] and rheumatoid arthritis [RA]).^1^^,^^2^ This results in diverse adverse effects, including gastrointestinal, hepatic, pulmonary, neurological, and cardiotoxic effects.^3^^,^^4^ Persistent clinical challenges include treatment-induced gut microbiota disruptions. This disruption affects drug efficacy and toxicity, making it difficult to reliably predict patient responses. Therefore, it is essential to understand the mechanisms underlying these phenomena.

Recent advances in next-generation sequencing (NGS) technologies have enhanced our understanding of the gut microbiota. Notably, the critical influence of the gut microbiota on the efficacy of chemotherapy agents has been highlighted.^5^^,^^6^ This has led to the emergence of the field of “pharmacomicrobiomics” to describe their interactions.^7^^,^^8^ Antimetabolite drugs interfere with nucleic acid synthesis, consequently altering the composition and function of the host microbiota. This altered microbial state influences therapeutic outcomes through diverse mechanisms, highlighting the bidirectional relationship between microbiota and drug activity. Furthermore, the microbiota plays critical roles in the pharmacokinetics and pharmacodynamics of antimetabolite drugs, profoundly influencing their efficacy and side effects.^9^ Consequently, predicting the efficacy of antimetabolite drugs via microbiota profiling or enhancing drug outcomes by modulating microbial communities has become an increasingly active area of research.

**Figure 1.**
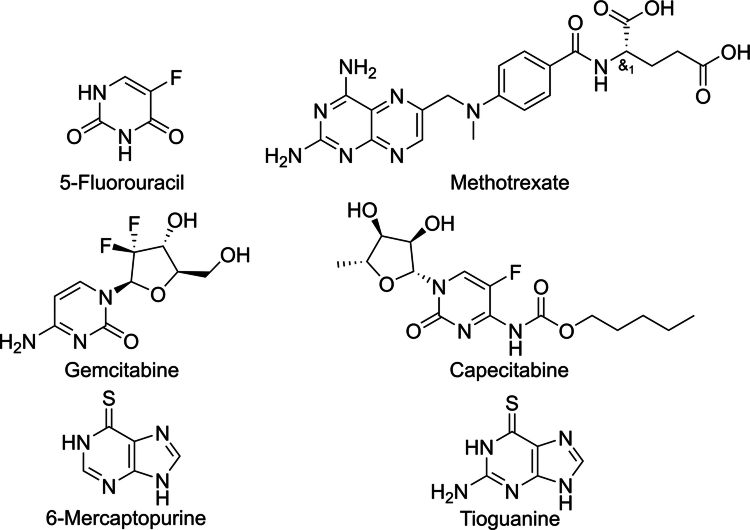
Structures of the antimetabolite drugs.

Although most studies focus on the relationship between individual drugs and gut microbiota, comprehensive reviews systematically elucidating the complex interactions between antimetabolite drugs and the gut microbiota are lacking. In this review, we focus on several commonly used antimetabolite drugs, including 5-FU, MTX, gemcitabine, capecitabine, 6-MP, and thioguanine (TG). Their structures are shown in Figure 1. This review summarizes the effects of antimetabolite drugs on gut microbiota composition and elucidates their underlying mechanisms. We explore how the microbiota and its metabolites influence drug efficacy, highlight potential microbial biomarkers for predicting treatment responses, and discuss recent strategies targeting microbiota modulation to enhance therapeutic outcomes.

## 2. Impact of antimetabolite drugs on the gut microbiota

Antimetabolites alter the gut microbiota composition and metabolic activity (Table 1 shows the impact of antimetabolite drugs on the gut microbiota). Although drug-specific effects vary, similar trends have been reported. These include reduced alpha-diversity (*α*-diversity, to measure species richness and evenness), a decreased *Firmicutes*/*Bacteroidetes* (F/B) ratio (indicative of dysbiosis, as these phyla comprise approximately 90% of gut bacteria),^10^ depletion of beneficial taxa (e.g., *Lactobacillus*), and enrichment of potentially pathogenic/proinflammatory taxa (e.g., *Proteobacteria*). These microbial shifts may contribute to adverse effects, such as mucositis and gastrointestinal toxicity. However, most studies focus on certain drugs, such as 5-FU, necessitating further investigation across a wider range of antimetabolites.

**Table 1.** Impact of antimetabolite drugs on the gut microbiota.

Chemotherapy	Microbiota feature		Effect	Reference
5-fluorouracil	Population	*α*-diversity↓	A reduction in *α*-diversity weakens the protective effect of beneficial bacteria, allowing pathogenic species to colonize.	[11‐20]
		F/B ratio↓	It stands for the imbalance in the ratio of *Firmicutes* to *Bacteroidetes*, which leads to the proliferation of pathogenic bacteria and gut microbiota dysbiosis.	[13,16,21‐24]
	Phylum	*Firmicutes*↓	A decrease in *Firmicutes* abundance downregulates the production of short-chain fatty acids and influences dietary energy absorption.	[12‐16,21‐23,25‐29]
		*Bacteroidetes*↑*	Changes remain controversial, with factors such as animal/disease model type, dietary composition, drug dosage, treatment duration, and environmental differences potentially contributing to the discrepancies.	[13,20‐23,27]
		*Bacteroidetes*↓*		[11,14,25,28‐33]
		*Proteobacteria*↑	An increase in *Proteobacteria* leads to the rise of intestinal pathogens, such as *Escherichia coli* and *Salmonella*.	[12,14,15,22,25,26,28,29,31,32]
		*Verrucomicrobia*↑	Its increase is generally believed to promote intestinal inflammation.	[11,13,25,31]
	Genus	*Bacteroides*↑*	Changes in *Bacteroides* abundance are also controversial.	[13,18,21,31,34,35]
		*Bacteroides*↓*		[11,36,37]
		*Lactobacillus*↓	A decrease in *Lactobacillus* populations is often associated with the proliferation of pathogenic bacteria and the disruption of gastrointestinal tract health.	[15,17,18,28,34,35,37‐39]
		*Verrucomicrobium*↑	Similar to *Verrucomicrobia*, *Verrucomicrobium* promotes intestinal inflammation.	[16,23]
Methotrexate	Population	*α*-diversity↑*	At low doses, Methotrexate increases *α*-diversity, while at high doses, it decreases *α*-diversity.	[40‐43]
		*α*-diversity↓*		[3,41,44‐46]
		F/B ratio↑*	At low doses, Methotrexate increases F/B ratio, while at high doses, it decreases F/B ratio.	[41,47]
		F/B ratio↓*		[4,41,44]
Methotrexate	Phylum	*Firmicutes*↑*	At low doses, Methotrexate increases the abundance of *Firmicutes*, while at high doses, it decreases that of *Firmicutes*.	[47]
		*Firmicutes*↓*		[44]
		*Bacteroidetes*↑*	At low doses, Methotrexate decreases the abundance of *Bacteroidetes*, while at high doses, it increases that of *Bacteroidetes*.	[44]
		*Bacteroidetes*↓*		[3,47]
	Family	*Ruminococcaceae*↓	A decrease in *Ruminococcaceae* results in impaired gut degradation functions.	[3,41,46,48,49]
	Genus	*Lactobacillus*↓	It’s associated with the proliferation of pathogenic bacteria and the disruption of gastrointestinal tract health.	[45,48,50]
		*Prevotella*↓	A reduction in *Prevotella* may indicate that the inflammation is alleviated.	[49,51,52]
	Species	*Bacteroides fragilis*↓	A decrease in *Bacteroides fragilis* may suggest the impairment of immune regulatory function and exacerbation of inflammation.	[3,49]
Gemcitabine	Population	*α*-diversity↑	Unknown.	[53,54]
	Phylum	*Firmicutes*↓	It downregulates the production of short-chain fatty acids and influences dietary energy absorption.	[55]
		*Bacteroidetes*↓	Unknown.	[55]
		*Proteobacteria*↑	It leads to the rise of intestinal pathogens, such as *Escherichia coli* and *Salmonella*.	[55]
		*Verrucomicrobia*↑	Its increase is generally believed to promote intestinal inflammation.	[55]
		*Actinobacteria*↓	The decrease in *Actinobacteria* may be associated with a reduction in the beneficial bacterium *Bifidobacterium*, leading to a weakened gut protective function and increased susceptibility to pathogenic bacteria.	[56]
	Species	*Akkermansia muciniphila*↑	It has a dual effect, improving metabolism, restoring intestinal barrier function, and alleviating inflammation, while also degrading mucins and exacerbating intestinal inflammation.	[55]
Capecitabine	Phylum	*Firmicutes*↓	It downregulates the production of short-chain fatty acids and influences dietary energy absorption.	[57]
		*Bacteroidetes*↑	Unknown.	[57]
		*Proteobacteria*↑	It leads to the rise of intestinal pathogens, such as *Escherichia coli* and *Salmonella*.	[58]
	Genus	*Bifidobacterium*↑	It leads to a weakened gut protective function and increased susceptibility to pathogenic bacteria.	[58]
Mercaptopurine	Population	*α*-diversity↓	It weakens the protective effect of beneficial bacteria, allowing pathogenic species to colonize.	[59]
	Phylum	*Firmicutes*↓	It downregulates the production of short-chain fatty acids and influences dietary energy absorption.	[59]
		*Proteobacteria*↑	It leads to the rise of intestinal pathogens, such as *Escherichia coli* and *Salmonella*.	[59]
	Genus	*Bacteroides*↑	Unknown.	[59]
		*Prevotella*↓	A reduction in *Prevotella* may indicate that the inflammation is alleviated.	[59]
	Species	*Akkermansia muciniphila*↑	It has a dual effect, improving metabolism, restoring intestinal barrier function, and alleviating inflammation, while also degrading mucins and exacerbating intestinal inflammation.	[59]
Tioguanine	Phylum	*Firmicutes*↑	Unknown.	[60]
		*Bacteroidetes↓*	Unknown.	[60]

Abbreviations: *α*-diversity: alpha-diversity; F/B: Firmicutes/Bacteroidetes; ↑: increased levels/index/ratio; ↓: reduced levels/index/ratio. Legend: Unknown means that the effect is still unknown. * means that the changes in the microbiota feature are controversial. Dotted lines divide the table into sections based on different drugs and the hierarchical levels of microbial species genera.

### 2.1. 5-Fluorouracil

The uracil analog 5-FU exerts anticancer effects by inhibiting TS and incorporating its metabolites into ribonucleic acid (RNA) and DNA. 5-FU is primarily used in the treatment of various malignancies, including CRC, head and neck, breast, gastric, and skin cancers.^61^ Major adverse effects include cardiotoxicity, gastrointestinal toxicity, and myelosuppression.

Several studies have shown that 5-FU treatment reduces gut microbial *α*-diversity ^11‐20^ and the F/B ratio.^13,16,21‐24^ At the phylum level, most studies report decreased *Firmicutes* abundance and increased *Proteobacteria* and *Verrucomicrobia* abundance. However, the reported changes in *Bacteroidetes* abundance remain inconsistent. At the genus level, a consistent finding is decreased *Lactobacillus* abundance. The specific details of these microbial shifts are summarized in Table 1.

Alpha-diversity (*α*-diversity) measures within-sample species richness and evenness.^62^ It is a crucial determinant of gut microbiome health and influences the therapeutic responses. The intestinal microbiota protects the host by competitively excluding pathogens through a mechanism known as colonization resistance.^63^ A reduction in *α*-diversity weakens this protective barrier, allowing pathogen overgrowth, lipopolysaccharide (LPS) release, host inflammation,^64^ and impaired drug efficacy, as demonstrated in 5-FU–treated CRC mouse models.^17^ Another important indicator is the F/B ratio, reflecting the relative abundance of two predominant gut phyla and correlates with microbial homeostasis. Associations between the F/B ratio and age,^65^ IBD,^66^ diabetes,^67^ and obesity^68^ underscore its relevance to the host immune and metabolic status.

Phylum-level analyzes reveal distinct microbial shifts. *Firmicutes*, gram-positive bacteria vital for intestinal homeostasis and short-chain fatty acid (SCFAs) production,^10^ typically decrease, potentially exacerbating 5-FU–induced mucositis.^16^ Conversely, the abundance of gram-negative phyla, such as *Proteobacteria* and *Verrucomicrobia*, often increases. Elevated levels of *Proteobacteria*, which are normally a minor component^69^ but contain pathogens, indicate dysbiosis and contribute to the development of intestinal disease.^70^ An increased *Verrucomicrobia* abundance is associated with intestinal inflammation.^25^ Findings regarding *Bacteroidetes*, gram-negative polysaccharide degraders, remain contradictory across studies and are likely confounded by variations in experimental conditions, such as the model, diet, or treatment protocol.

Similar to the observations for *Bacteroidetes*, changes in the genus *Bacteroides* are inconsistent across studies, potentially reflecting its high variability among individuals.^69^ Another key genus is *Lactobacillus* (phylum *Firmicutes*), which ferments carbohydrate into lactic acid. These common residents of the human oral and gastrointestinal tracts are widely used as probiotics and exert crucial protective functions. A decline in *Lactobacillus* population is frequently associated with the overgrowth of pathogenic bacteria.

In addition to compositional changes, 5-FU significantly affects gut microbial function and gene expression. It alters genomic mutation profiles and markedly reduces G:C to T:A transversions. Evolutionary pressure can be assessed via the dN/dS ratio, which compares non-synonymous (dN) to synonymous (dS) substitution rates. This ratio appeared to stabilize (dN/dS = 80.1%) in most of the Kyoto Encyclopedia of Genes and Genomes (KEGG) functional modules.^36^ This widespread stability likely reflects purifying selection (dN/dS < 1), which preserves the core microbial functions under 5-FU treatment. This contrasts with neutral evolution (dN/dS ≈ 1, mutations are only affected by random genetic drift) and positive selection (dN/dS > 1, beneficial mutations are retained).^71^ Nevertheless, certain gene sets involved in cell proliferation and protein secretion showed elevated dN/dS ratios. This implies positive selection for adaptive strategies, such as enhanced proliferation or specific secretion, to survive 5-FU exposure. Additionally, 5-FU enriches microbial genes are related to the nicotinamide adenine dinucleotide (NAD) salvage pathway in patients. Activation of this pathway suppresses cancer cell autophagy and enhances survival. This finding suggests a possible mechanism by which the gut microbiota modulates therapeutic outcomes.

### 2.2. Methotrexate

MTX is an anti-folate immunosuppressant, with folate being its primary target.^72^ MTX inhibits folate metabolism by competitively inhibiting DHFR, thereby blocking the *de novo* synthesis of purines and pyrimidines. MTX exerts dose-dependent effects.^72^ with low-dose MTX used for treating autoimmune diseases such as psoriasis, IBD, and RA^73^ and high-dose MTX used for cancers such as leukemia, lymphoma, and osteosarcoma.^74^ Common adverse effects of MTX include gastrointestinal, bone marrow, cardiac, neurologic, renal, and hepatic toxicities.^3^^,^^4^

MTX also exerts a dose-dependent effect on the gut microbiota (Table 1). Low doses generally increase *α*-diversity and the F/B ratio, illustrated by improved diversity in patients with RA^40^ and increased F/B ratio in gnotobiotic mice.^47^ On the other hand, high doses reduce diversity, induce inverse phylum shifts, and cause mucositis in healthy rats.^44^

Furthermore, MTX altered specific gut microbial taxa. Reported effects include reduced abundances of the family *Ruminococcaceae*, genera *Lactobacillus* and *Prevotella*, and the species *Bacteroides fragilis* (Table 1).

Such changes have functional implications, as the family *Ruminococcaceae* (phylum *Firmicutes*), comprising gram-positive bacteria, degrades resistant starch into SCFAs ^75^ and its depletion may impair gut degradation capabilities. At the genus level, *Prevotella* (phylum *Bacteroidetes*), a gram-negative bacterium, is enriched in patients with RA and associated with intestinal inflammation.^76^^,^^77^ Reduced *Prevotella* levels following MTX treatment may signify increased RA inflammation. Finally, the gram-negative species *Bacteroides fragilis* (phylum *Bacteroidetes*) plays a key role in immune modulation and suppression of inflammation;^78^ thus, its reduction might exacerbate inflammation.

MTX alters gut microbiota composition by targeting folate metabolism. Approximately 86% of the human gut microbial species are folate auxotrophs depending on external sources such as diet or cross-feeding, whereas only a minority (approximately 13%) are capable of *de novo* synthesis.^79^ This widespread dependence indicates that MTX-induced inhibition of folate metabolism leads to a broad suppression of the microbiota. For instance, the growth *Prevotella*, which requires external folate,^80^ is consequently stunted by MTX.^81^ Studies have demonstrated that folate-deficient diets decrease both *α*- and *β*-diversity in the fecal microbiota of healthy humans.^82^ This corroborates the role of folate inhibition in MTX-induced microbiota alterations.

### 2.3. Gemcitabine and capecitabine

Gemcitabine is a deoxycytidine nucleoside analog and prodrug. After entering the human body, it is activated by deoxycytidine kinase to form gemcitabine di- and triphosphates. Gemcitabine diphosphate subsequently inhibits ribonucleotide reductase and gemcitabine triphosphate competes with deoxycytidine triphosphate for incorporation into DNA, ultimately inhibiting DNA synthesis and inducing apoptosis. Gemcitabine is widely used to treat ovarian, bladder, non-small cell lung, pancreatic, and breast cancer.^83^ Although gemcitabine can cause myelosuppression and gastrointestinal and pulmonary toxicity, its toxicity is generally milder than that of other pyrimidine antagonists.^84^ The primary challenges associated with gemcitabine include enzymatic deamination, rapid systemic clearance, and resistance.^85^

Gemcitabine increases *α*-diversity, reduces *Firmicutes*, *Bacteroidetes*, and *Actinobacteria* and increases *Proteobacteria, Verrucomicrobia,* and *Akkermansia muciniphila*. The specific microbial changes are listed in (Table 1).

Examining specific taxa provides further information regarding their functional impact. The phylum *Actinobacteria*, a group of gram-positive microbes found widely in nature, is a crucial source of metabolites, including most antibiotics and bioactive substances utilized by humans.^86^ Consequently, a reduction in *Actinobacteria* could signify a loss of beneficial genera, such as *Bifidobacterium*, thereby potentially compromising gut protection and increasing vulnerability to pathogens. *A. muciniphila* (gram-negative bacterium belonging to the phylum *Verrucomicrobia*) exhibits context-dependent functions. It promotes the host health by enhancing metabolism, reinforcing the gut barrier, and reducing inflammation.^87^ Conversely, it can degrade mucin, potentially worsening intestinal inflammation, particularly in malnourished hosts.^88^ Despite these potential microbial interactions, studies directly assessing the effects of gemcitabine on the human gut microbiota remain limited.

Capecitabine, an oral prodrug of 5-FU, is converted into 5-FU by thymidine phosphorylase, thereby exerting its antitumor effects.^89^ It has shown varying degrees of efficacy and acceptable tolerability in several cancers, including prostate, renal cell, ovarian, pancreatic, metastatic breast, and CRC cancers.^90^ Common toxicities of capecitabine include gastrointestinal and cardiovascular toxicities, as well as hand-foot syndrome.^91^

The effects of capecitabine on the gut microbiota included a decrease in *Firmicutes* at the phylum level, an increase in *Bacteroidetes* and *Proteobacteria*, and an increase in *Bifidobacterium* at the genus level. However, some studies have reported no significant effect of capecitabine on the gut microbiota,^92^ warranting further research (Table 1).

*Bifidobacterium,* a gram-negative bacterium from the *Actinobacteria* phylum, is a probiotic that can inhibit pathogenic bacteria and improve gut barrier function. It plays a regulatory role in various intestinal diseases including allergies, IBD, and cancer.^93^^,^^94^ Increased *Bifidobacterium* levels are beneficial for gut health.

### 2.4. Mercaptopurine and thioguanine

The purine analogs 6-mercaptopurine (6-MP) and TG are important antimetabolites. 6-MP, often administered via its prodrug azathioprine (AZA) for potentially enhanced bioavailability,^95^ inhibits purine nucleotide biosynthesis and subsequent DNA synthesis. TG, a guanine analog, requires activation by hypoxanthine-guanine phosphoribosyl transferase to form cytotoxic nucleotides that are incorporated into and damage DNA, similar to the downstream effects of 6-MP.^96^ Both agents are used to treat acute lymphoblastic leukemia,^97^ and 6-MP is used to treat lymphoma and RA. Common primary toxicities include bone marrow suppression, gastrointestinal disturbances, and hepatic and renal dysfunction.^98^

6-MP reduces *α*-diversity, *Firmicutes and Prevotella,* and increases *Proteobacteria*, *Bacteroides* and *A. muciniphila* ^59^. The effect of TG on the gut microbiota is mainly at the phylum level, increasing *Firmicutes* and decreasing *Bacteroidetes* ^60^. However, research on these drugs is currently limited.

## 3. Influence of the gut microbiota and its metabolites on antimetabolite drug efficacy and toxicity

Emerging evidence highlights the influence of the gut microbiota on antimetabolite drug efficacy and toxicity. Although the general TIMER framework (Translocation, Immunomodulation, Metabolism, Enzymatic degradation, and reduced diversity/ecological variation) proposed by Alexander et al.^99^ describes chemotherapy-microbiota interactions, it may not fully capture the specificity of antimetabolites targeting nucleic acid biosynthesis. Therefore, we propose a framework that encompasses absorption, metabolism, distribution, excretion, endogenous metabolite production, immune modulation, and apoptotic pathway modulation, to delineate the specific mechanisms underlying gut microbes regulation of antimetabolite outcomes.

### 3.1. Microbe-induced changes to drug pharmacokinetics

#### 3.1.1. Microbe-induced drug absorption

The gut microbiota regulates intestinal permeability by modulating the integrity of tight junctions and thickness of the intestinal mucus layer, ultimately influencing the absorption of pharmacological agents.

Antimetabolite drugs can cause gut microbiota dysbiosis exacerbating damage to tight junction proteins among intestinal epithelial cells and disrupting the cell surface mucus layer, thereby increasing intestinal epithelial permeability.^26^ In contrast, probiotic interventions strengthen intercellular tight junctions, increase mucus layer thickness, and decrease permeability.^100^ The state of intestinal tight junctions and the mucus layer profoundly impacts drug diffusion, epithelial absorption, and overall bioavailability. Studies have confirmed that targeted modulation of tight junctions can facilitate paracellular drug transport.^101^ In contrast, a thickened mucus layer inhibits effective drug delivery.^102^

However, few studies have specifically investigated the effects of these structural changes on drug absorption, as most research has focused on the status of tight junctions and the mucus layer as indicators of chemotherapy-induced intestinal barrier disruption and mucositis.^14^ Thus, the influence of the gut microbiota on antimetabolite drug absorption remains incompletely understood.

#### 3.1.2. Microbe-induced drug metabolism

The gut microbiota contains millions of protein-coding genes capable of metabolizing diverse nutrients and altering drug pharmacokinetics.^7^ Increasing evidence indicates that bacteria-derived enzymes can influence the response and toxicity of antimetabolite drugs.^99^

**Figure 2.**
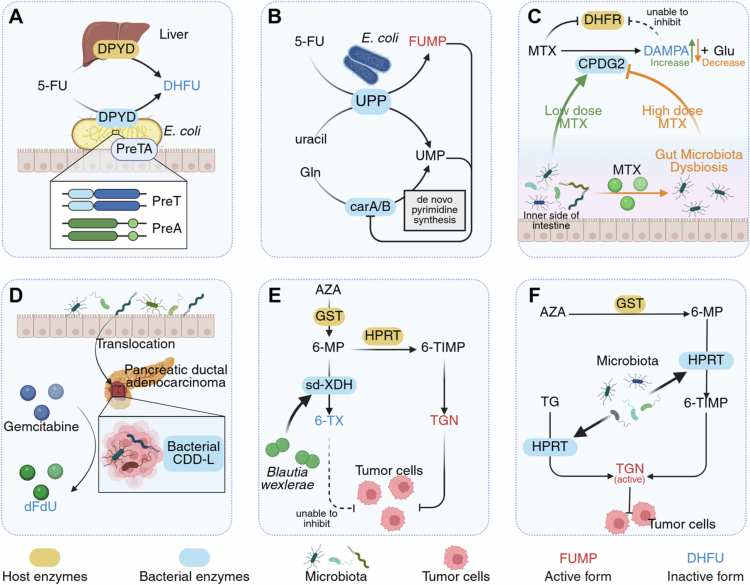
Mechanisms by which the gut microbiota alters drug metabolism and affects the activity of antimetabolite drugs. A The preTA operon in *E. coli* can express enzymes similar to DPYD in the host liver and degrade 5-FU to the inactive metabolite DHFU. B The UPP enzyme in *E. coli* metabolizes 5-FU to FUMP, which blocks the *de novo* pyrimidine synthesis pathway and inhibits tumor growth. **C** At low doses, MTX is metabolized by CPDG2 in the gut microbiota to the inactive metabolite DAMPA, but at higher doses, MTX-induced dysbiosis downregulates CPDG2, delaying DAMPA generation. D A CDD-L enzyme present in the gut microbiota translocated to pancreatic tumors can degrade gemcitabine to the inactive metabolite dFdU. E *Blautia wexlerae*-derived sd-XDH degrades AZA or 6-MP to the inactive metabolite 6-TX. F The gut microbiota expresses enzymes similar to those of the host HPRT, facilitating the conversion of 6-MP, AZA, and TG into the active metabolite TGN. 5-FU, 5-fluorouracil; 6-MP, mercaptopurine; 6-TIMP, 6-thioinosine monophosphate; 6-TX, 6-thioxanthine; AZA, azathioprine; carA/B, carbamoyl phosphate synthetase; CDD, cytidine deaminase; CPDG2, carboxypeptidase glutamate 2; DAMPA, 2, 4-diamino-*N*-10-methylpteroic acid; DHFR, dihydrofolate reductase; DHFU, dihydrofluorouracil; dFdU, 2',2'-difluorodeoxyuridine; DPYD, dihydropyrimidine dehydrogenase; FUMP, 5-fluorouridine monophosphate; Gln, glutamine; Glu, glutamic acid; GST, glutathione S-transferase; HPRT, hypoxanthine phosphoribosyltransferase; MTX, methotrexate; sd-XDH, selenium-dependent xanthine dehydrogenase; TGN, 6-thioguanine nucleotide; UMP, uridine monophosphate; upp, uracil phosphoribosyltransferase.

The *preTA* operon in the gut microbiota encodes dihydropyrimidine dehydrogenase (DPYD), that reduces the bioavailability and chemotherapeutic efficacy of 5-FU by metabolizing it to the inactive metabolite dihydrofluorouracil (DHFU) (Figure 2A). In the mammalian liver, DPYD metabolizes 5-FU to inactive DHFU. Recent findings have shown that the *preTA* operon in *Escherichia coli* encodes DPYD and metabolizes 5-FU, thereby reducing the bioavailability and efficacy of oral 5-FU treatment in mice.^103^ Moreover, cell-free supernatants from *preTA*-overexpressing *E. coli* significantly reduce the inhibitory effect of 5-FU on tumor cells.^103^ The *preTA* operon is commonly found in the human gut, particularly in *Proteobacteria* and *Firmicutes* ^103^. The genome of *Anaerostipes hadrus* also harbors the *preTA* operon and can metabolize 5-FU into DHFU; however, it also contains *hydA*, which encodes bacterial dihydropyrimidinase, enabling the metabolism of DHFU into *α*-fluoro-*β*-ureidopropionic acid.^104^ However, the *hydA* gene in *E. coli* is located further from the *preTA* operon; therefore, 5-FU metabolism is halted at DHFU.^103^^,^^104^

A recent prospective cohort study used metagenomic sequencing to corroborate the interaction between *preTA* and oral fluoropyrimidines, including the 5-FU prodrug capecitabine,^105^ revealing that chemotherapy enriches pyrimidine metabolism genes (such as *preTA* and *upp*) in the human gut microbiome—explaining why gastrointestinal toxicity often appears early in treatment and later subsides; and that oral fluoropyrimidine toxicity is microbiome-dependent, with bacterial *preTA* mitigating adverse effects. Furthermore, a predictive model based on baseline *preTA* levels demonstrated high accuracy in assessing patient treatment toxicity. Collectively, these studies indicate that bacterial *preTA* has significant clinical potential as a crucial biomarker and interventional target for regulating drug efficacy and toxicity.

Uracil phosphoribosyltransferase (UPP) in the gut microbiota converts 5-FU to 5-fluorouridine monophosphate (FUMP), thereby increasing chemotherapy efficacy (Figure 2B). A study using *Caenorhabditis elegans* suggested that 5-FU and its prodrug derivative, 5-fluoro-2′-deoxyuridine (FUDR), act via the regulation of ribonucleotide rather than DNA metabolism.^106^ Both 5-FU and FUDR are metabolized to FUMP in *E. coli* via the pyrimidine salvage pathway, which is encoded by an upregulated gene.^106^ Uridine monophosphate (UMP) blocks *de novo* pyrimidine synthesis in both bacteria and hosts by inhibiting the carA/B enzymes. It has been hypothesized that FUMP, a structural analog of UMP, may exert a similar effect; however, this has not been experimentally validated. By converting 5-FU and FUDR into FUMP, microbial enzymes may block de novo pyrimidine synthesis, thus inhibiting tumor growth.^106^

Carboxypeptidase glutamate 2 (CPDG2) in the gut microbiota converts MTX into inactive 2, 4-diamino-*N*-10-methylpteroic acid (DAMPA), thereby reducing MTX efficacy and mitigating its toxicity (Figure 2C). CPDG2 inactivates MTX and its metabolite 7-OH-MTX by cleaving the terminal glutamate residues, yielding DAMPA and 7-OH-DAMPA, respectively.^41^^,^^107^ The essential role of the microbiota in this conversion is confirmed by the absence of DAMPA in germ-free or antibiotic-treated mice.^108^ *p*-aminobenzoyl-glutamate hydrolase found in *E. coli* is also capable of this reaction.^109^ As DAMPA does not inhibit DHFR, this microbial activity reduces the effects of MTX. Clinically, CPDG2 has been approved as an antidote for delayed MTX clearance.^110^

Importantly, MTX-induced microbiota alterations downregulate CPDG2, potentially resulting in delayed MTX detoxification (Figure 2C). At low MTX doses, 7-OH-MTX is excreted within 12 h, whereas DAMPA is excreted between 12 and 48 h. However, at higher MTX doses, the excretion of MTX and DAMPA is delayed to 48 h.^41^ High-dose MTX-induced alterations in the gut microbiota may reduce CPDG2 activity, thereby delaying MTX detoxification into DAMPA.^41^ Specifically, high-dose MTX may decrease the abundance of bacterial families, such as *Prevotellaceae, Anaeroplasmataceae, Ruminococcaceae,* and *Lactobacillaceae*, that are positively correlated with DAMPA excretion and likely contribute to CPDG2 production. These alterations could impair MTX clearance by decreasing overall CPDG2 activity.^41^

Folylpolyglutamate synthase (FPGS) in the gut microbiota can convert MTX into MTX-PG, although its effect on the therapeutic outcomes of MTX may be minimal. Certain gut bacteria can add glutamate to MTX via FPGS to generate MTX-PG,^111^ that displays a greater affinity for its target proteins compared to MTX. However, it has a lower affinity for the reduced folate transporter and proton-coupled folate transporter, which complicates its transport. Therefore, MTX-PGs produced by the gut microbiota are unlikely to significantly affect the therapeutic efficacy of MTX.^112^

Cytidine deaminase (CDD) in intratumoral microorganisms found in pancreatic ductal adenocarcinoma (PDAC) converts gemcitabine into an inactive metabolite, thereby reducing its efficacy (Figure 2D). Geller et al. reported that the long isoform of bacterial CDD (CDD-L) from *Mycoplasma hyorhinis* in human dermal fibroblasts can convert gemcitabine into the inactive 2′,2′-difluorodeoxyuridine, thus mediating gemcitabine resistance.^113^ Similarly, CDD from *Enterococcus faecium* mediates gemcitabine resistance in gallbladder cancer.^114^ Consistently, antibiotic treatment or treatment with CDD-deficient *E. coli* significantly improved the antitumor response to gemcitabine in mice. Geller et al. further investigated intratumoral bacteria in human PDAC,^113^ revealing that the proportion of tumors containing bacteria (76%) was significantly higher than that in the normal pancreas (15%) and the most common species detected was *Gammaproteobacteria*. The authors hypothesized that these bacteria migrate retrogradely from the duodenum to the pancreas. Pushalkar et al. further confirmed this hypothesis using bacterial translocation experiments, which revealed that fluorescently-labeled *Enterococcus faecalis* migrated into the pancreas of mice,^115^ suggesting direct influence of gut bacteria on the pancreatic microenvironment. The gut and pancreatic microbiomes of patients with PDAC exhibited similar community profiles, with enrichment of *Proteobacteria*. Recent studies have suggested that fecal microbiota transplantation (FMT) can modulate the characteristic microbiome enrichment observed in patients with PDAC.^116^ This modulation creates a favorable tumor microenvironment and potentially improves patient prognosis. Similar to the gut microbiota, a recent study demonstrated that oral bacteria also contribute to gemcitabine resistance in pancreatic cancer via CDD.^117^ Oral bacteria may invade the pancreatic tissue through hematogenous spread or gastrointestinal translocation, leading to gemcitabine degradation. This study emphasized that the enzymatic function of CDD is independent of isoform length, contrasting with an earlier study that demonstrated that the long isoform is fully functional, whereas the short isoform retains only partial functionality.^113^

Gut microbial selenium-dependent xanthine dehydrogenase (sd-XDH) inactivates 6-MP, the active metabolite of AZA, by converting it to 6-thioxanthine (6-TX), thereby reducing the therapeutic effectiveness of AZA in IBD (Figure 2E). Specifically, *Blautia wexlerae*-encoded sd-XDH converts 6-MP to 6-TX, which decreases 6-thioinosine-monophosphate (6-TIMP), a precursor of the active drug form, thioguanine nucleotide (TGN), ultimately, contributing to AZA treatment failure in IBD.^118^

Gut microbial hypoxanthine phosphoribosyltransferase (HPRT) converts TG into its active form, TGN (Figure 2F). Recent evidence suggests that TG exerts therapeutic effects in IBD through bacterial metabolism, independent of the adaptive immune system. Although HPRT is considered critical for this conversion, TG has shown efficacy in HPRT-deficient mice without systemic immunosuppression.^60^ *In vitro* studies have demonstrated that *E. coli*, *E. faecalis*, and *Bacteroides thetaiotaomicron* can produce TGN from TG, implicating the involvement of bacterial HPRT. Strategies to enhance TG efficacy may involve modulating the intestinal microbiota or optimizing drug delivery to increase the colonic residence time.^119^ Notably, *Bacillus subtilis*, which also encodes HPRT, increases 6-TIMP levels, thereby rescuing AZA treatment failure induced by *Blautia wexlerae* in IBD.^118^

#### 3.1.3. Microbe-induced drug distribution

Gut microbiota and its metabolites can regulate the distribution of drugs by modulating drug transporters on tumor cell surfaces, thereby affecting the efficacy and toxicity of antimetabolite drugs.

**Figure 3.**
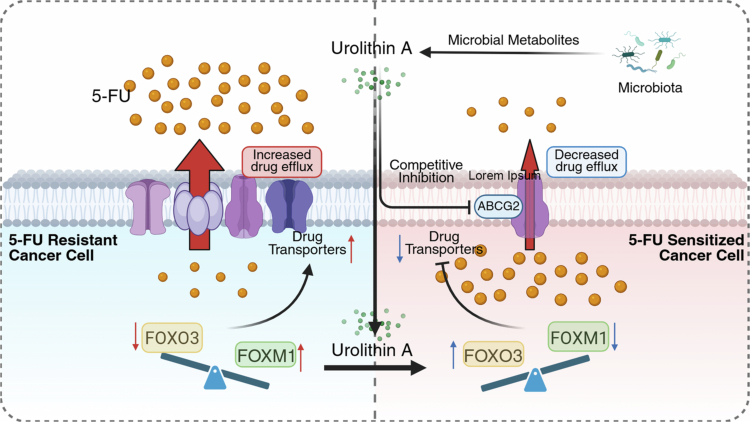
Mechanisms by which urolithin A downregulates drug transporters. The metabolite urolithin A produced by the gut microbiota regulates the FOXO3-FOXM1 axis, leading to reduced expression of drug transporters on the tumor surface and decreased efflux of 5-FU. Additionally, urolithin A can competitively inhibit the drug transporter ABCG2, further reducing the efflux of 5-FU. 5-FU, 5-fluorouracil; FOXM1, forkhead box M1; FOXO3, forkhead box O3.

Urolithins downregulate various drug transporters, reduce the efflux of 5-FU, and enhance drug efficacy. Urolithin is a gut microbiota metabolite derived from the dietary polyphenols ellagitannins and ellagic acid, divided into urolithin A (UroA) and urolithin B (UroB).^120^ Urolithins, especially UroA, demonstrate broad antitumor activities.^121^ UroA and its structural analog, UAS03, downregulate the expression of drug transporters (MDR1, BCRP, MRP2, and MRP7), thereby reducing the efflux of 5-FU and sensitizing cancer cells.^122^ Specifically, forkhead box (FOX) proteins play crucial roles, with FOXM1 and FOXO3 acting as upstream regulators of drug transporters. FOXM1 enhances the expression of the drug transporter ABCC10 and mediates 5-FU resistance. Conversely, FOXO3 directly inhibits FOXM1 and antagonizes FOXM1 function by competing for the same target genes.^123^ Therefore, elevated FOXM1 and decreased FOXO3 expression are associated with resistance to cancer therapy. UroA/UAS03 reduces the expression of drug transporters by regulating the FOXO3-FOXM1 axis, thus sensitizing CRC cells to 5-FU (Figure 3).

Additionally, ATP-binding cassette (ABC) transporters are crucial for 5-FU transport during chemotherapy.^124^ Their subfamilies B (e.g., ABCB1), C (e.g., ABCC1/2/3), and G (e.g., ABCG2) mediate drug efflux. UroA reverses 5-FU resistance by inhibiting ABCG2^125^ (Figure 3). In contrast, butyrate—a gut microbiota-derived SCFA and histone deacetylase inhibitor with antitumor activity^126^—is potentially associated with 5-FU resistance via ABCG2. Chronic butyrate exposure downregulates its transporter, sodium-coupled monocarboxylate transporter 1 (SMCT1), causing butyrate resistance in CRC cells.^127^ Butyrate-resistant CRC cells (HCT-116/BR) show cross-resistance to 5-FU, possibly due to their high ABCG2 expression.^128^^,^^129^ Notably, *Lactobacillus plantarum*-derived metabolites can overcome this dual resistance by upregulating SMCT1.^127^

#### 3.1.4. Microbe-induced drug excretion

The gut microbiota indirectly influences the excretion of antimetabolites, primarily by modulating the systemic burden of the active drug and its metabolites. For instance, gut microbes metabolize MTX into the non-toxic DAMPA via CPDG2. The enzymatic activity of microbial CPDG2 directly impacts the elimination rates of both 7-OH-MTX and DAMPA.^41^ Thus, microbial metabolic inactivation of antimetabolites is frequently and intimately tied to overall drug excretion.

Additionally, the gut microbiota can subject drugs to repeated metabolism through enterohepatic circulation, thereby affecting hepatic excretion. In contrast, its influence on renal excretion is more indirect, largely affecting the excretory load of the kidney based on the systemic concentrations of the parent compound and its derivatives.

### 3.2. Microbe-induced endogenous metabolite production

Microbiota-derived endogenous metabolites—including amino acids, nucleotides, and regulatory factors—modulate host responses to antimetabolite drugs via multiple pathways.

**Figure 4.**
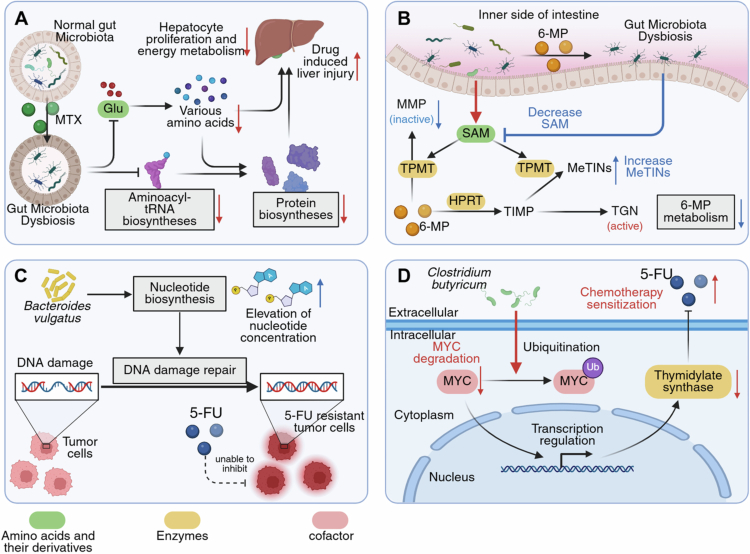
Mechanisms by which the gut microbiota regulates various endogenous metabolites and affects the efficacy of antimetabolite drugs. **A** MTX-induced dysbiosis inhibits the biosynthesis of aminoacyl-tRNA, downregulates the metabolism of various amino acids, such as glutamic acid, affects hepatocyte proliferation and energy metabolism, and mediates drug-induced liver injury. **B** 6-MP-induced dysbiosis downregulates SAM concentrations and inhibits TPMT activity, thereby affecting the metabolism of 6-MP. **C** *Bacteroides vulgatus*-mediated nucleotide biosynthesis promotes DNA damage repair in tumor cells, conferring resistance to 5-FU. **D** *Clostridium butyricum* promotes the ubiquitination and degradation of MYC, leading to the downregulation of thymidylate synthase. 5-FU, 5-fluorouracil; 6-MP, mercaptopurine; Glu, glutamic acid; HPRT, hypoxanthine phosphoribosyltransferase; MeTINs, methylthio-6⁃thioinosine phosphates; MMP, 6-methylmercaptopurine; MTX, methotrexate; SAM, S-adenosylmethionine; TGN, thioguanine nucleotide; TIMP, thioinosine monophosphate; TPMT, thiopurine S-methyltransferase; Ub, ubiquitin.

Gut microbiota enhances MTX-induced liver injury by influencing aminoacyl-tRNA biosynthesis. Given the critical role of the liver in amino acid homeostasis and the association between disrupted amino acid metabolism and liver toxicity,^130^ modulation of aminoacyl-tRNA, which is essential for protein synthesis, is significant. Specifically, *Ruminococcus* and *Lactobacillus* promote aminoacyl-tRNA biosynthesis, whereas *Collinsella* and *Streptococcus* inhibit it. MTX perturbs these microbial populations, suppressing aminoacyl-tRNA biosynthesis and downregulating amino acids, such as glycine, L-aspartic acid, and L-valine, ultimately leading to reduced protein synthesis and drug-induced liver injury (DILI)^48^ (Figure 4A).

Furthermore, the gut microbiota contributes to liver damage by modulating glutamic acid metabolism. Glutamic acid is a multifunctional amino acid that functions as a neurotransmitter and participates in the metabolism of several other amino acids. *Lactobacillus*, *Streptococcus*, *Enterococcus*, and other bacteria are associated with glutamic acid metabolism. MTX reduces the abundance of gut microbes by downregulating glutamic acid and its associated amino acid metabolic pathways, impairing hepatocyte proliferation and energy metabolism, ultimately leading to DILI^48^ (Figure 4A).

Tryptophan (TRP) metabolism is another critical pathway regulated by gut microbiota. Research has shown that the probiotic *Weissella paramesenteroides* WpK4 alleviates 5-FU-induced intestinal mucositis^131^ by producing and catabolizing TRP and generating endogenous metabolites such as indole-3-acetic acid (IAA) and tryptamine. IAA acts as a ligand and activates the aryl hydrocarbon receptor (AhR), which translocates from the cytoplasm to the nucleus where it forms a heterodimeric complex with the AhR nuclear translocator. This complex initiates the transcription of specific target genes and plays a crucial role in local immune response. Prebiotic dried ginger essential oil also modulates TRP metabolism in the gut microbiota,^132^ generating metabolites including IAA, 5-hydroxyindole-3-acetic acid, and oxoadipic acid, thereby mitigating 5-FU-induced mucositis. This study proposed an IAA-based AhR/interleukin (IL)22/signal transducer and activator of transcription 3 (STAT3) signaling axis, where AhR activation by IAA induces the expression of downstream cytokines, such as IL-22 and IL-17,^133^ with IL-22 subsequently activating the STAT3 pathway. This pathway is essential for regulating and maintaining intestinal barrier integrity while suppressing apoptosis-related processes.^134^ In addition to IAA, other TRP metabolites influence chemotherapy efficacy. Under high-salt diet conditions, the gut bacterial TRP metabolism affects FOLFOX efficacy via immunomodulation.^135^ The TRP metabolites indole, skatole (SK), indole propionic acid (IPA) and indole-3-carboxaldehyde significantly increased IL-1β and TNF-*α* production in macrophages. In contrast, tryptophan, tryptamine, and indole-3-lactic acid significantly decreased them. Interestingly, although IAA promoted the efficacy of FOLFOX upon long-term exposure, it inhibited its efficacy during short-term treatment. This study further demonstrates that SK suppression and IPA enhancement potentiate the therapeutic effects of FOLFOX, although the underlying mechanisms remain unclear.

Gut microbiota modulates 6-MP metabolism via S-adenosylmethionine (SAM) production. SAM serves as a ubiquitous methyl donor that is critical for cellular methylation.^136^ Treatment with 6-MP induces gut dysbiosis and reduces SAM levels in the cecum and plasma, thereby impairing the stability and activity of thiopurine S-methyltransferase, an enzyme dependent on SAM for 6-MP methylation.^59^ Consequently, 6-MP metabolism shifts are characterized by decreased formation of the primary methylated metabolite 6-methylmercaptopurine and increased accumulation of secondary methylated products, methylthio-6-thioinosine phosphates^59^ (Figure 4B). Bacteria in the genus *Alistipes* are implicated in SAM dysregulation.^59^

The gut microbiota also promotes FOLFOX resistance by increasing SAM concentration.^137^ *Desulfovibrio desulfuricans* and its metabolites elevate serum SAM levels in the gut. As a crucial intracellular methyl donor, SAM interacts with methyltransferase-like 3 (METTL3), increasing the number of catalyzed RNA methylation modification reactions. Upregulation of METTL3 via the JAK2-STAT3 pathway leads to the expression of pro-oncogenic genes and confers FOLFOX resistance in CRC cells. METTL3 silencing can counteract this resistance, thereby restoring chemotherapeutic efficacy. However, the impact of SAM on tumors remains controversial. One perspective suggests that, at low concentrations, SAM upregulates METTL3 and promotes tumor progression,^137^ whereas at high concentrations, it induces cell death and autophagy in tumor cells.^138^ The effect of SAM is likely dominated by the former mechanism under the influence of gut microbiota, rather than exogenous supplementation.

Gut microbes can diminish the efficacy of 5-FU by increasing endogenous nucleotide production, which enhances DNA repair in tumors. For instance, *Bacteroides vulgatus* levels are significantly elevated in patients with locally advanced rectal cancer who respond poorly to neoadjuvant chemoradiotherapy.^20^ This bacterium promotes resistance by stimulating the *de novo* nucleotide biosynthesis pathway, thereby facilitating tumor DNA repair and protecting cancer cells from chemotherapy-induced damage^20^ (Figure 4C). Consistent with this mechanism, the administration of exogenous nucleosides or *B. vulgatus* in mouse colorectal tumor models significantly reduced the therapeutic effect of 5-FU.

Host ribonucleotide and deoxyribonucleotide metabolism, which is regulated by microbial communities, critically influences 5-FU efficacy.^139^ Studies on *Caenorhabditis elegans* show that bacteria affect 5-FU by participating in ribonucleotide metabolism, facilitating the activation of 5-FU (e.g., FUMP/FUTP) and by modulating the host deoxyribonucleotide pool, thereby altering downstream effects of 5-FU TS inhibition, including DNA damage.^139^ The role of ribonucleotide metabolism in 5-FU activation has been supported by multiple studies,^106^^,^^139^ albeit with different interpretations. Earlier work highlighted UPP-mediated FUMP production and pyrimidine synthesis inhibition,^106^ whereas Scott et al. emphasized the subsequent conversion to FUTP, leading to RNA damage and cell death.^139^ Regarding the deoxyribonucleotide pathway, Scott et al. proposed that bacterial modulation of host deoxyribonucleotide levels induces DNA damage and bacteria-dependent autophagy, thereby altering 5-FU response.^139^ In contrast, *Bacteroides vulgatus* promotes DNA repair via nucleotide biosynthesis.^20^ However, Scott et al. suggested that a bacterial deoxyribonucleotide imbalance could influence 5-FU efficacy independent of DNA repair.^139^ Collectively, these results suggest that bacterial deoxyribonucleotide metabolism potentially affects 5-FU outcomes through both autophagy and DNA damage/repair pathways.

The gut microbiota-derived secondary bile acid, deoxycholic acid (DCA) enhances the efficacy of FOLFOX in CRC.^140^ DCA mediates this enhancement by upregulating the AOX3/Cyp26b1 pathway through activation of *Ugt1a6b*, a key component of the enterohepatic circulation of bile acids. *Ugt1a6b* encodes the UDP-glucuronosyltransferase enzyme, which plays a pivotal role in bile acid enterohepatic circulation. The combination of DCA and FOLFOX increased the mRNA expression of *Ugt1a6b* and other genes associated with enterohepatic circulation. This promotes the glucuronidation of bile acids and improves enterohepatic cycling, thereby enhancing FOLFOX reabsorption and increasing its therapeutic efficacy. Further investigations are required to determine whether other bile acids exert similar effects.

*Clostridium butyricum* influences 5-FU efficacy by modulating regulatory factors. This bacterium enhances proteasome-mediated ubiquitination and subsequent degradation of the MYC oncogene, which in turn promotes the transcription of thymidylate synthase (TS). TS is a key target enzyme of 5-FU, and its overexpression often causes 5-FU resistance.^141^ Therefore, *C. butyricum*-mediated MYC degradation reduces TS expression, which sensitized tumors to 5-FU treatment^142^ (Figure 4D), thereby leveraging the known tumor-suppressive effect of MYC inactivation.^143^

### 3.3. Microbe-induced immune modulation

Growing evidence highlights the capacity of the gut microbiota to modulate host immunity.^144^ In the context of antimetabolite chemotherapy, this microbial influence affects treatment efficacy by modulating tumor and inflammatory immunity, as well as treatment toxicity by altering inflammatory responses underlying side effects such as enteritis.^145^ The microbiota regulates both the innate and adaptive immune systems. Importantly, this immunomodulation is not straightforward potentiation or inhibition but a complex, bidirectional interaction often described as a “double-edged sword.”

#### 3.3.1. Innate immunity

The gut microbiota interacts with various pattern recognition receptors (PRRs) to regulate the innate immune response, including toll-like receptors (TLRs) and nucleotide-binding oligomerization domain (NOD)-like receptors (NLRs).

TLRs are a family of PRRs that recognize conserved microbial structures (pathogen-associated molecular patterns) and host-derived danger signals (damage-associated molecular patterns).^146^ Continual interactions between gut microbes and the epithelium result in physiological low-grade inflammation. In contrast, chemotherapy-induced dysbiosis disrupts this homeostasis, often hyperactivating TLR pathways and promoting inflammatory damage.^147^ Canonical TLR signaling typically proceeds via the myeloid differentiation primary response gene 88 (*MyD88*)-dependent pathway, culminating in nuclear factor-κB (NF-κB) activation and proinflammatory cytokine secretion.^148^

**Figure 5.**
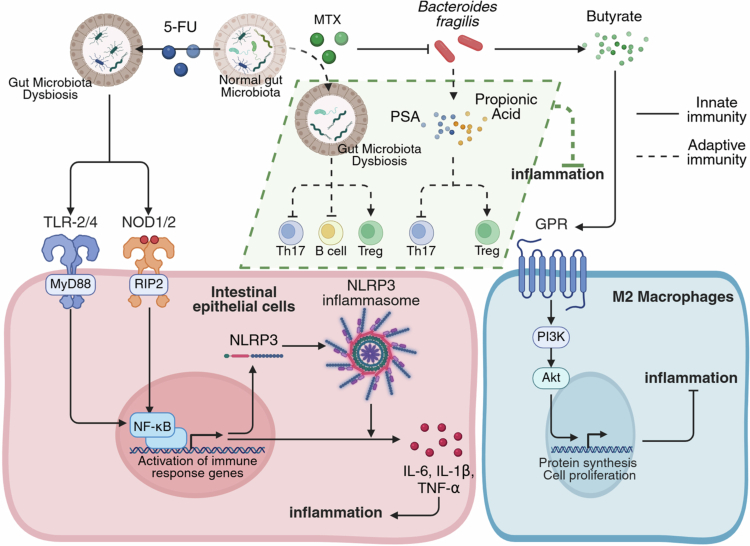
Mechanisms by which the gut microbiota influences drug efficacy through immune regulation. In terms of innate immunity, the microbiota activates the TLR-2/4 and NOD1/2 receptors, promoting the expression of the NLRP3 inflammasome and various inflammatory cytokines. The metabolite butyrate also stimulates M2 macrophage proliferation through GPR receptors. In adaptive immunity, the microbiota and their metabolites regulate the proportions of various immune cells. 5-FU, 5-fluorouracil; GPR, G protein-coupled receptor; MTX, methotrexate; NLRP-3, NOD-like receptor thermal protein domain associated protein 3; NOD, nucleotide binding oligomerization domain; PSA, polysaccharide A; TLR, Toll-like receptor.

The roles of TLR2 (which detects gram-positive bacteria) and TLR4 (which detects gram-negative bacteria) are well established in intestinal inflammation.^149^ 5-FU-induced dysbiosis activates both TLR2/MyD88/NF-κB and TLR4/MyD88/NF-κB signaling pathways in rats, elevating inflammatory cytokines such as IL-1β, IL-6, and TNF-*α*^18^^,^^26^ (Figure 5). Specific bacterial taxa correlate with this signaling: certain gram-positive genera (*Aerococcus*, *Blautia*, *Roseburia*) are inversely associated with TLR4 activity, whereas gram-negative genera (*Bacteroides*, *Parabacteroides*, *Parasutterella*) show positive correlations and may exacerbate inflammation.^21^ Conversely, probiotic interventions such as *Akkermansia* supplementation can alleviate inflammation by downregulating TLR2 and inhibiting NF-κB signaling.^150^

Microorganisms also influence immune responses via NLRs. Unlike TLRs, which detect extracellular or endosomal ligands, NLR proteins NOD1 and NOD2 function as cytosolic PRRs that recognize bacterial peptidoglycans within host cells.^151^ Upon ligand recognition, NOD1 and NOD2 oligomerize to activate receptor-interacting protein 2 (RIP2), which triggers downstream signaling cascades involving both NF-κB and mitogen-activated protein kinase (MAPK) pathways, ultimately driving the transcription of immune-related genes and exacerbating RA.^152^^,^^153^ 5-FU-induced microbiota dysbiosis upregulates NOD1/2 expression, activating NOD/RIP2/NF-κB signaling and altering the secretion of inflammatory cytokines including IFN-*γ*, TNF-*α*, IL-1β, and IL-6,^30^^,^^154^ Figure 5.

NOD-like receptor thermal protein domain-associated protein 3 (NLRP-3), anotheran important member of the NLR family, also influences the immune response. NLRP-3 forms a multiprotein signaling complex (NLRP-3 inflammasome) under pathogenic stimulation.^155^ Once activated, it induces the production of proinflammatory cytokines such as IL-1β and IL-18, thereby causing inflammation and facilitating tissue and organ regeneration.^156^ NLRP-3 inflammasome is highly expressed in intestinal tissues of mice with 5-FU-induced mucositis, where sustained PRR stimulation by harmful bacteria activates the NLRP-3 inflammasome, thereby increasing TNF-*α*, IL-6, IL-1β, and IL-18 levels and exacerbating inflammation^157^ (Figure 5). Beneficial microbes such as *A. muciniphila* and its outer membrane protein Amuc_1100 reduce the activation of NLRP-3 inflammasomes, alleviating 5-FU-induced mucositis in mice.^158^

The gut microbiota also modulates innate immunity by influencing macrophage activation and polarization.^159^ For instance, *B. fragilis* promotes anti-inflammatory M2 macrophages, critical for regulating inflammatory diseases.^160^ Notably, *B. fragilis* abundance diminishes following MTX treatment in both preclinical models and patients with RA.^3^^,^^49^ MTX efficacy in collagen-induced arthritis models is dependent on *B. fragilis,* with its transplantation restoring MTX activity in microbiota-depleted mice.^49^ This bacterium appears to enhance the efficacy of MTX by increasing the M2 macrophage population^49^ (Figure 5), potentially via butyrate production. *B. fragilis* elevates butyrate levels, and butyrate alone can restore MTX efficacy in such models.^49^ Butyrate promotes M2 proliferation and dampens inflammation via G protein-coupled receptor mediated phosphatidylinositol 3-kinase (PI3K)/protein kinase B (Akt) signaling.^161^ In contrast, colonization with *Bacteroides wexlerae* promotes inflammatory macrophages and reduces AZA efficacy in a mouse model of colitis,^118^ emphasizing species-specific effects on drug response.

#### 3.3.2. Adaptive immunity

Adaptive immunity, including both humoral and cell-mediated pathways, is also modulated by the intestinal microbiota. In the context of antimetabolite drug therapy, microbial regulation mainly affects cell-mediated immune responses.

The gut microbiota influences T-cell proliferation and differentiation, potentially enhancing the anti-inflammatory efficacy of MTX. MTX functions through multiple host mechanisms (including nucleotide synthesis inhibition, adenosine release, reactive oxygen species [ROS], and cytokines),^162^ and also targets microbial pathways, reducing immune activation.^51^^,^^163^ Transferring MTX-altered microbiota into germ-free mice reduces activated T cell populations, including T helper 17 (Th17) cells, suggesting that bacteria such as *Prevotella copri* and *Collinsella aerofaciens* mediate adaptive immune suppression after MTX treatment^51^ (Figure 5). Clinically, MTX combined with glucocorticoids helps restore the Th17/regulatory T cell (Treg) balance in patients with early RA by modulating the microbiota,^42^ thereby targeting Th17-driven inflammation in RA.^164^

*B. fragilis* promotes Treg proliferation and suppresses adaptive immunity via metabolites. Its immunoregulatory molecule, polysaccharide A, inhibits IL-17 production while inducing CD4 + T cell differentiation into IL-10-producing Foxp3 + Tregs, ameliorating colitis in animal models.^165^ *B. fragilis* also utilizes SCFAs for immunoregulation in conditions such as IBD.^166^ The YCH46 strain of *B. fragilis* produces propionic acid, which increases Tregs and decreases Th17 cells, thereby reducing inflammation ^167^ (Figure 5).

*Parabacteroides distasonis* also influences T cell responses. A study on high-dose MTX for primary central nervous system lymphoma revealed a correlation between gut *P. distasonis* abundance and prognosis.^168^ This bacterium may act via the gut-brain axis; its higher abundance is associated with increased CD8+ T cell infiltration in the cerebrospinal fluid, a marker of favorable outcomes. Further analysis indicated a positive correlation between *P. distasonis* abundance and plasma betaine-valerate levels. Thus, *P. distasonis* can slow disease progression by indirectly promoting CD8+ T cell infiltration into the cerebrospinal fluid and tumor tissue.

### 3.4. Microbe-induced apoptotic pathway modulation

Antimetabolite drugs can induce apoptosis in tumor cells by inhibiting DNA biosynthesis and repair as well as through various apoptotic pathways. For example, 5-FU can activate the death receptor tumor necrosis factor receptor 1 by upregulating TNF-*α*, leading to the initiation of the extrinsic apoptotic pathway, which causes the activation of caspase-3 and caspase-8, culminating in cell apoptosis.^23^ Studies have demonstrated that gut microbiota can significantly alter the apoptosis-inducing therapeutic efficacy of antimetabolite drugs through multiple apoptotic pathways.

**Figure 6.**
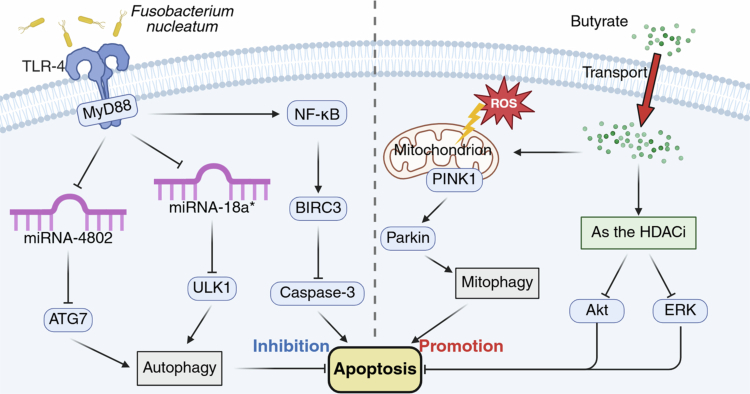
Mechanisms by which the gut microbiota regulates apoptosis pathways. *Fusobacterium nucleatum* promotes the expression of the autophagy signaling elements ATG7 and ULK1 and inhibits apoptosis through the activation of TLR-4. This bacterium also upregulates the anti-apoptotic protein BIRC3 via TLR-4, further suppressing apoptosis. The microbiota metabolite butyrate induces mitochondrial oxidative stress, promoting mitophagy, which can increase apoptosis. Additionally, butyrate functions as an HDAC inhibitor, further facilitating apoptosis. HDACi, histone deacetylase inhibitor; ROS, reactive oxygen species; TLR, Toll-like receptor.

*Fusobacterium nucleatum*, a pathogenic bacterium, mediates 5-FU resistance by suppressing apoptotic pathways in colon cancer cells.^169^^,^^170^ *F. nucleatum* can specifically activate the TLR4 and MYD88 immune signaling pathways in tumor cells, inhibiting specific microRNAs and increasing the expression of autophagy signaling elements such as ULK1 and ATG7. This shift in signaling from apoptosis to autophagy ultimately overwhelms the 5-FU-induced apoptotic pathway, thereby mediating resistance to 5-FU treatment.^170^ Additionally, BIRC3, a member of the inhibitor of apoptosis protein family, inhibits apoptosis by directly inhibiting the caspase cascade.^171^ *F. nucleatum* can upregulate BIRC3 in CRC cells via the TLR-4/NF-kB pathway, leading to the suppression of tumor cell apoptosis and resistance to 5-FU chemotherapy ^169^ (Figure 6).

*F. nucleatum* promotes anti-apoptotic effects in colon cancer cells by releasing extracellular heptose-related metabolites.^172^ ADP-heptose (ADP-H) and heptose-1, 7-bisphosphate are produced by gram-negative bacteria that trigger the ALPK1/TIFA pathway, leading to NF-κB activation.^173^ *F. nucleatum* releases an ADP-H-related molecule into its microenvironment, which exhibits the biological characteristics of ADP-H, an ALPK1 ligand, and potently activates NF-κB in intestinal epithelial cells via the ALPK1/TIFA/TRAF6 pathway. Furthermore, *F. nucleatum* releases butyrate, which synergizes with ADP-H in the ALPK1-dependent activation of NF-κB, thereby increasing the expression of the inflammatory cytokine IL-8 and two anti-apoptotic genes, *BIRC3* and *TNFAIP3*, ultimately promoting CRC cell survival and reducing the chemosensitivity to 5-FU *in vitro*.

In contrast, the gut microbial metabolite butyrate potentiates 5-FU efficacy against CRC through distinct pro-apoptotic mechanisms. Butyrate preferentially accumulates within CRC cells owing to their reliance on glycolysis (Warburg effect),^174^ unlike normal colonocytes, which utilize butyrate as an energy source.^175^ As an endogenous histone deacetylase inhibitor, intracellular butyrate modulates the Akt/ERK and SMAD3 signaling pathways,^126^^,^^176^ enhancing apoptosis and 5-FU sensitivity.^177^ Furthermore, sodium butyrate (NaB) triggers ROS-mediated mitophagy linked to apoptosis via the PINK1/Parkin pathway^178^ (Figure 6). Consistent with these effects, the combination of 5-FU and NaB yielded synergistic antiproliferative effects *in vitro* and *in vivo*. This combination therapy can also foster a healthier gut microbial composition by enriching beneficial taxa (e.g., *Bacteroidetes*, *Ligilactobacillus*, and butyrate/acetate producers), potentially alleviating 5-FU toxicity. The chemosensitizing properties of butyrate may be extended to drugs such as gemcitabine for pancreatic cancer, although the specific mechanisms require further investigation.^53^

A recent study further explored the mechanisms underlying NaB-induced apoptosis, demonstrating that NaB enhances the 5-FU-mediated growth inhibition and apoptosis of gastric cancer cells by directly downregulating TS expression.^179^ Specifically, NaB downregulated the expression of key genes involved in DNA synthesis (*TYMS* and *TK1*), DNA replication (*MCM4* and *RRM2*), cell cycle progression (*CCNE1* and *CCNA2*), cell proliferation (*FOXM1* and *c-Myc*), and stress responses (*SIRT1* and *DNAJA1*), leading to cell cycle arrest and suppression of DNA replication. Notably, *TYMS* encodes TS, the target enzyme of 5-FU, whereas TK1 is critical for the recovery of dTMP synthesis. Downregulation of both enzymes attenuates tumor cell resistance to 5-FU. Furthermore, NaB induces apoptosis by disrupting mitochondrial energy metabolism and ATP synthesis. Moreover, sodium propionate exerts similar effects and mechanisms as those of NaB, including the modulation of TS, FOXM1, and MCM4.^180^ These findings suggest that the sodium salts of gut microbiota-derived SCFAs possess pro-apoptotic effects and potentiate the efficacy of antimetabolite drugs. However, this hypothesis requires further validation through additional research.

## 4. Microbial biomarkers for predicting the response to antimetabolite drugs

Emerging evidence indicates that gut microbiota profiles can predict patient responses to antimetabolite drugs such as MTX in RA (Table 2). A key study demonstrated that the baseline gut microbial taxonomic composition and functional potential in treatment-naive patients with RA were significantly correlated with the subsequent clinical response to MTX.^181^ Good responders (GR) exhibited lower microbial diversity than poor responders (PR). The PR group was characterized by a higher F/B ratio and enrichment of specific taxa (e.g., *Euryarchaeota*, *Escherichia/Shigella*), while the GR group showed higher relative abundances of *Bacteroides* and *Prevotella*. Functionally, patients with PR had enriched pathways related to MAPK signaling, DNA replication, and fatty acid degradation. In contrast, patients with GR had diminished pathways involving LPS metabolism and folate biosynthesis. By leveraging these metagenomic features, a random forest machine learning model successfully predicted MTX response status with 80% accuracy in an independent validation cohort (*n* = 21).^181^

**Table 2.** Microbial biomarkers for predicting the response to antimetabolite drugs.

Chemotherapy	Model	Response	Gut microbiota	Microbial gene functions	Reference
FOLFOX	Low-set rectal cancer patients	GR	**Before treatment:** *Clostridium ramosum*; **After treatment:** *Clostridiales bacterium* 1.7.47 FAA	**Before treatment:** Tetrahydrofolate, ornithine, and ubiquinol biosynthesis and degradation pathways	[19]
		PR	**Before treatment:** *Megamonas rupellensis* and *Coprobacter fastidiosus*; **After treatment:** *Parvimonas micra*, *Bacteroides clarus*, *Porphyromonas uenonis*, *Solobacterium moorei*, and *Coprobacter fastidiosus*	**Before treatment:** Aromatic amino acid and deoxyribonucleotide biosynthesis; **After treatment:** Petroselinate biosynthesis, glycolysis, and ester metabolism	
nCRT*	LARC patients	GR	**Before treatment:** *Avsunviroidae*, *Bromoviridae*, *Flaviviridae*, *Virgaviridae*. *Viruses_noname*, *Paraprevotella*, *Pelamoviroid*, *Trichovirus*, *Cucumovirus*, *Tobamovirus*, *Viruses_noname*; **After treatment:** *Negativicutes*, *Selenomonadales*, *Actinomycetaceae*, *Burkholderiaceae*, *Veillonellaceae*, *Actinomyces*, *Megasphaera*	**After treatment** Mixed acid fermentation and guanosine diphosphate-mannose biosynthesis	[20]
		PR	**Before treatment:** *Prevotella* **After treatment** *Deltaproteobacteria*, *Desulfovibrionales*, *Burkholderiales*, *Desulfovibrionaceae*, *Burkholderiales*, *Anaerostipes*, *Bilophila*	**After treatment** Biosynthesis of nucleotides, including purF, which encodes a rate-limiting enzyme in de novo purine biosynthesis, and five genes related to pyrimidine nucleotide synthesis	
MTX	new-onset RA patients	GR	**Before treatment** *α*-diversity↓, F/B ratio↓ *Bacteroides*, *Prevotella*↑ *Clostridiales*, *Ruminococcus*↓	**Before treatment** Lipopolysaccharide biosynthesis and either carbohydrate/vitamin metabolism or biosynthetic pathways, most notably folate biosynthesis	[181]
MTX	new-onset RA patients	PR	**Before treatment** *α*-diversity↑, *Firmicutes*↑, *Bacteroidetes*↓, F/B ratio↑ *Euryarchaeota phylum*, *unclassified Clostridiales*/*Clostridiales incertae* sedis XIII family, and *Escherichia/Shigella genera*↑	**Before treatment** The MAPK signaling pathway, DNA replication, fatty acid degradation, and ABC transporters	[181]
MTX	Psoriasis patients	GR	**Before treatment:** *α*-diversity↓ **After treatment:** *Species Bacteroides vulgatus* and *family Veillonellaceae*↑; *Species Bacteroides caccae* and *family Erysipelotrichaceae*↓	**After treatment:** Levels in the super pathway for biosynthesis of arginine and polyamine, the pathway for degradation of purine nucleotides, and the pathway for biosynthesis of phosphatidylglycerol↑	[182]
		PR	**Before treatment:** *α*-diversity↑, *class Bacilli*, *order Lactobacillales*, *family Leuconostocaceae*, *family Burkholderiales noname*, *genus Burkholderiales noname*, *species Burkholderiales bacterium_1_1_47*, *species Gemella sanguinis*, and *species Bacteroides feces*; **After treatment:** *class Clostridia*, *order Clostridiales*, *family Streptococcaceae*, and *family Ruminococcaceae*	**After treatment** Methylerythritol phosphate level↑ Methylglyoxal degradation↓	
MTX	RA patients	GR	*Prevotella* enterotype	Glycan biosynthesis and metabolism	[183]
		PR	*Bacteroides* enterotype	Vitamins biosynthesis and peptidoglycan biosynthesis	
MTX	RA patients	GR	*Prevotella copri*, *Haemophilus pittmaniae*, *Megamonas unclassified*, *Alistipes indistinctus*, *Parabacteroides distasonis*, *Bifidobacterium bifidum*	Biosynthesis of several kinds of amino acids	[184]
		PR	*Subdoligranulum unclassified*, *Ruminococcus champanellensis*, *Anaerotruncus unclassified*, *Dorea longicatena*, *Ruminococcus obeum*, *Eubacterium siraeum*, *Eubacterium hallii*, *Parabacteroides goldsteinii*, *Bacteroides intestinalis*	Pyruvate fermentation to acetate and lactate	

Abbreviations: α-diversity: alpha-diversity; ABC: ATP-binding cassette transporter; F/B: Firmicutes/Bacteroidetes; GR, good response; LARC, locally advanced rectal cancer; MAPK: mitogen-activated protein kinase; MTX, methotrexate; nCRT: neoadjuvant chemoradiotherapy; PR, poor response; RA, rheumatoid arthritis; ↑: increased levels/index/ratio; ↓: reduced levels/index/ratio. Legend: FOLFOX includes leucovorin, oxaliplatin, and 5-fluorouracil; nCRT*: Here includes oxaliplatin, capecitabine, and radiotherapy; Before/After treatment: characteristics of the gut microbiota and gene function before/after treatment. Dotted lines divide the table into sections based on different research studies and response groups.

A distinct predictive approach categorized patients with RA into enterotypes based on the dominant bacterial genera.^183^ Functional gene analysis revealed the enrichment of glycan synthesis and metabolism pathways in the *Prevotella*-dominant enterotype (RA E1), in contrast to the enrichment of vitamin and peptidoglycan synthesis pathways in the *Bacteroides*-dominant enterotype (RA E2). Clinically, patients belonging to the RA E1 enterotype demonstrated significantly better responses to MTX treatment than those in the RA E2 group.^183^

Gut microbial metabolic activity is associated with MTX efficacy in psoriasis.^182^ Post-MTX treatment, the GR group showed reduced organic and fatty acid levels, whereas the PR group showed elevated carbohydrates and amino acids, indicating that differential metabolism is associated with treatment outcomes. Mirroring the findings in RA,^181^ higher microbial diversity was observed in the PR group, potentially signifying greater resistance to MTX-induced changes. Specific taxa, including *Bacilli*, *Lactobacillales*, and unclassified *Burkholderiales* members, were enriched in the PR group. Distinct microbial functional signatures were also apparent; GR microbiomes showed increased activity in arginine/polyamine biosynthesis, purine degradation, and phosphatidylglycerol synthesis pathways, but decreased mannan degradation. Conversely, notable pathway alterations in PR microbiomes were mainly limited to methylerythritol phosphate and methylglyoxal degradation.^182^

Microbial metabolites are emerging predictors of sensitivity to chemotherapy. For example, differences in the gut microbiota between high-fat diet-induced obese and lean mice influence tumor responses to gemcitabine/paclitaxel, with lean mice showing better responses. This effect was microbiota dependent, as demonstrated by FMT experiments,^185^ which revealed distinct microbial metabolic outputs with obese mice showing enrichment in queuosine biosynthesis and lean mice favoring SAM metabolism. Queuosine promotes chemoresistance, which may involve upregulation of peroxiredoxin 1. In contrast, the antitumor metabolite SAM enhances chemosensitivity, with SAM administration improving treatment responses in obese mice.^185^ Similarly, in diabetic mouse models, the enrichment of menaquinol-producing bacteria was correlated with chemoresistance,^186^ likely due to its antioxidant capacity, which protects against drug-induced ROS. These findings highlight the regulation of chemotherapy outcomes by microbiota-derived metabolites such as queuosine, SAM, and menaquinol.

## 5. Strategies to improve antimetabolite drug response via the gut microbiota

An increasing number of chemical adjuvants and interventions, namely prebiotics, probiotics, synbiotics, postbiotics, dietary intervention, and FMT, have been developed to enhance the efficacy of antimetabolites and mitigate their toxicity **(Table S1)**.

### 5.1. Supplementation with prebiotics, probiotics, synbiotics, or postbiotics

#### 5.1.1. Prebiotics

Prebiotics, as defined by the International Scientific Association of Probiotics and Prebiotics, are substrates selectively utilized by host microorganisms to confer health benefits.^187^ Numerous prebiotics have been identified that may offer benefits during antimetabolite drug therapy. These are primarily derived from plant and animal sources, encompassing carbohydrate molecules (e.g., astragalus polysaccharides and fructooligosaccharides (FOSs)) and noncarbohydrate compounds (e.g., cryptotanshinone and casuarinin). Synthetic prebiotic mixtures, such as certain fiber blends, represent a smaller category **(Table S1)**.

Natural carbohydrate prebiotics (polysaccharides and oligosaccharides) derived from diverse sources modulate the gut microbiota to improve antimetabolite therapy outcomes. Several studies have demonstrated their benefits, particularly in the mitigation of 5-FU toxicity. Oyster polysaccharide (CHP) and alginate oligosaccharide (AOS) both alleviate 5-FU–induced enteritis by enriching beneficial bacteria such as *Akkermansia*, increasing SCFA production, enhancing gut barrier integrity (AOS via MLCK signaling), and reducing inflammation (CHP via TLR signaling, AOS via TLR4/MyD88/NF-κB inhibition and apoptosis modulation).^24^^,^^26^ Polysaccharides from *Astragalus*, *Poria*, and *Albuca bracteata* showed similar potential **(Table S1)**. FOS, alone or with arginine, specifically target 5-FU–induced mucositis by boosting SCFAs, preserving barrier function, and promoting beneficial genera such as *Bacteroides* and *Lactobacillus* ^188^^,^^189^.

In addition to mitigating toxicity, prebiotics such as xylan can also be used for drug delivery.^190^ Common drug delivery strategies include liposomes, polymeric nanoparticles, lipid nanoparticles, inorganic nanoparticles, dual targeting nanoparticles, and exosomes.^191^ Xylan–stearic acid nanoparticles (SCXN) carrying capecitabine improved drug delivery to CRC tumors and enhanced survival in mice compared to free drugs.^192^ This system simultaneously modulated antitumor immunity (favoring dendritic cell maturation and CD8+ T cell responses over Tregs) and reshaped the gut microbiome toward a beneficial state (enriching *Clostridia*, *Lachnospiraceae*, and *Akkermansia*; suppressing *Desulfovibrio*; and boosting SCFAs).^192^ These effects highlight its multifaceted therapeutic potential.

A recent study developed 5-FU–loaded liposomes by combining the prebiotic xylan derivative Sxy with 1, 2-dipalmitoylphosphatidy-lethanolamine, a homolog of *A. muciniphila* active phospholipid. This delivery system enhanced CRC treatment efficacy via a mechanism similar to that of SCXN.^193^

In addition to carbohydrates, non-carbohydrate natural extracts can modulate the gut microbiota to enhance antimetabolite therapy. For example, cryptotanshinone (CTS), derived from *Salvia miltiorrhiza*, ameliorates 5-FU–induced colitis in mouse models by regulating microbial lipid metabolism that involves shifting specific bacterial populations (e.g., *Muribaculaceae* and *Lactobacillus*) and normalizing host lipid markers.^194^ CTS can also activate the PI3K/Akt pathway to exert positive effects.^195^ Another *Salvia* compound, dihydrotanshinone (DHTS), combats 5-FU mucositis by restoring microbiota balance, enriching *Akkermansia*, and suppressing systemic inflammatory markers such as IL-6 and TNF-*α* ^196^. Patchouli alcohol, from *Pogostemon cablin*, supports intestinal barrier integrity, partly by influencing microbiota structure and inhibiting TLR2/MyD88/NF-κB signaling, thereby preventing enteritis.^18^ Curcumin has also demonstrated promising prospects for treating ulcerative colitis.^197^ Other non-carbohydrate compounds such as berberine,^12^^,^^34^^,^^198^ casuarinin,^199^ and saikosaponin-A^38^ also leverage microbiota interactions to improve antimetabolite outcomes **(Table S1)**.

#### 5.1.2. Probiotics

Probiotics are live microorganisms that, when administered in adequate quantities, confer health benefits to the host ^200^ and can markedly influence outcomes during chemotherapy, including 5-FU and MTX treatments. Specific strains, such as *L. casei* var. *rhamnosus* and *L. reuteri* DSM 17938 mitigated 5-FU mucositis by modulating the microbiota balance.^201^ *Pediococcus pentosaceus* PP34 combats 5-FU toxicity by reducing oxidative stress and inflammation, concurrently altering gut microbial composition (e.g., enriching *Akkermansia*).^202^ *A. muciniphila* specifically protects against MTX intestinal injury and is linked to the maintenance of gut homeostasis and stem cells via Wnt signaling and SCFA production.^203^ The yeast *Saccharomyces boulardii* (CNCM I-745) exerts broad effects, improving bone/joint health, reducing inflammation in MTX-treated arthritis models,^204^ and alleviating 5-FU mucositis by limiting inflammation and restoring gut permeability.^205^

Probiotic mixtures also show promise: *Lactobacillus/Bifidobacterium* combinations regulated inflammation in 5-FU–treated CRC models (via NF-κB inhibition and mucin preservation),^206^ and *L. casei* Shirota/*B. breve* combinations enhanced antitumor immunity during gemcitabine/cisplatin treatment via the gut-tumor axis in bladder cancer models.^54^ A controlled clinical trial demonstrated that for psoriasis patients, supplementing MTX therapy by adding probiotics (*Bifidobacterium longum, L. acidophilus,* and *Enterococcus Capsules*) helped maintain intestinal barrier integrity more effectively than MTX alone.^207^ Serum levels of calprotectin and zonulin did not substantially increase in the combination group, unlike those in the MTX-only control group. A novel “intestinal–vaginal” synergistic drug delivery strategy combines oral *B. longum subsp. longum* NCU-05 with intravaginal *Lacticaseibacillus crispatus* NCU-28.^208^ This strategy enhanced the anticancer efficacy of 5-FU by activating the p53 signaling pathway and upregulating pro-apoptotic protein expression. It simultaneously restored intestinal and vaginal microbial homeostasis, thereby mitigating inflammation-related tissue damage at both sites.

#### 5.1.3. Synbiotics

Defined as a mixture of live microorganisms and substrate(s) selectively utilized by host microorganisms that confer health benefits to the host,^209^ synbiotics offer strategies to counteract chemotherapy complications. One example involves encapsulating specific *Bifidobacterium*, *Lactobacillus*, *Streptococcus*, and *Clostridium* strains in resistant starch; this synbiotic markedly reduced 5-FU–induced inflammation (cytokines, NF-κB signaling) while preserving microbial abundance and composition, thus mitigating dysbiosis.^27^ Another study used a combination of several *Lactobacillus* and *Bifidobacterium* strains with prebiotics (inulin and lactoferrin) to treat gemcitabine/nab-paclitaxel–induced dysbiosis in a pancreatic cancer model. This synbiotic increases microbial richness and SCFA levels, consequently reducing chemotherapy side effects and cancer-associated stromatogenesis.^210^ A multicenter randomized clinical trial included 61 patients with esophageal cancer receiving neoadjuvant chemotherapy (including 5-FU).^211^ This trial found that patients experienced a considerable reduction in adverse events when supplemented with synbiotics (containing *L. casei* Shirota and galacto-oligosaccharides) compared to the control group. This beneficial effect is attributed to the ability of synbiotics to enhance the abundance of beneficial gut bacteria and elevate the concentrations of SCFAs, specifically acetic and propionic acids, thereby mitigating the incidence of chemotherapy-induced febrile neutropenia.

#### 5.1.4. Postbiotic

Postbiotics, defined as preparations of inanimate microorganisms and/or their components that confer health benefits to the host,^212^ can potentially augment chemotherapy outcomes. For example, *L. rhamnosus*–derived postbiotics show promise: the cell-free supernatant from the strain GG displays selective anticancer activity and enhances 5-FU sensitization,^213^ whereas an inactivated preparation of the strain CGMCC1.3724 mitigates 5-FU–induced mucosal damage via mechanisms involving structural preservation, reduced inflammation, enhanced mucus production, and increased Treg populations.^214^ Microbial metabolites also function as postbiotics. For instance, UroB possesses potent immunomodulatory properties including boosting natural killer cell activity, inhibiting Tregs, downregulating PD-L1 expression, upregulating antigen presentation machinery (HLA-B and TCR), and enhancing overall antigen presentation. In combination with capecitabine, UroB regulates the gut microbiota and suppresses CRC tumor growth in mice.^215^

### 5.2. Dietary intervention

Dietary intervention to modify the gut microbiota can increase the efficacy of antimetabolite drugs. These interventions include dietary restriction (DR), fiber-rich diet, and ketogenic diet.

The benefits of DR on the efficacy of antimetabolite drugs remain controversial. Previous studies demonstrated that DR may benefit the prognosis of patients undergoing chemotherapy.^216^ Research has shown that 30% DR for two weeks before chemotherapy markedly improves overall survival in both young and old mice treated with 5-FU.^217^ DR prevents the loss of lysozymes and increases the abundance of *Lactobacillus*, thereby inhibiting the translocation of opportunistic pathogens into the gut. This effect was more prominent in older mice.^217^ Given that elderly patients often exhibit an increased abundance of opportunistic pathogens in their feces, DR holds considerable potential for improving the safety of and tolerance to chemotherapy in older individuals.^217^ Moreover, 30% DR for two weeks markedly improved the survival rates after exposure to a lethal dose of MTX. DR alleviates intestinal inflammation by increasing the abundance of *Lactobacillus* and preserving the number of basal crypt proliferating cell nuclear antigen-positive cells, thus protecting intestinal stem cell function.^45^ However, another study did not replicate these findings. It revealed that although 48 h of DR before treatment slowed epithelial proliferation and increased microbial diversity and richness, it did not affect the abundance of *Lactobacillus*. Furthermore, the beneficial effects of DR are overshadowed by its negative impact on body weight, resulting in a failure to effectively alleviate MTX-induced gastrointestinal mucositis.^218^ Therefore, exploring more rational DR strategies may be crucial for optimizing the effects of DR.

A fiber-rich diet in mice undergoing 5-FU treatment promoted the production of microbial metabolites and reduced neuroinflammation.^219^ This dietary intervention substantially altered the gut microbiota composition. It increased the abundance of *Bacteroidaceae* and *Akkermansiaceae* that correlated with increased propionate production, reduced astrocyte density, and subsequent alleviation of neuroinflammation.^219^ Furthermore, a whey-based diet containing medium-chain triglycerides (MCTs) has demonstrated beneficial effects against MTX-induced gastrointestinal damage.^220^ The readily absorbed MCTs provided rapid energy and facilitated the renewal and repair of intestinal epithelial cells. This dietary approach also modified the gut microbiota structure and activity, leading to an increase in branched-chain fatty acid levels and a reduction in MTX-induced gastrointestinal mucositis.^220^

### 5.3. FMT

FMT is used to directly alter a recipient’s gut microbiota to normalize its composition and derive therapeutic benefits.^221^ Clinically, FMT has been used to treat recurrent *C. difficile* infections, irritable bowel syndrome, and IBD, among other conditions.^222^ Importantly, the safety of FMT remains a challenge, with common adverse events including vomiting, abdominal pain, diarrhea, and infection.^223^ Screening of healthy donors is key to the success of FMT.^224^ Recent findings suggest that FMT can directly manipulate the gut microbiota and improve chemotherapy-induced mucositis. For example, FMT improved intestinal mucosal damage in a mouse model of colon cancer treated with FOLFOX chemotherapy (5-FU, oxaliplatin, etc.).^31^ Specifically, FMT reduced the severity of diarrhea and intestinal mucositis, inhibited the TLR/MyD88/NF-κB signaling pathway and apoptosis, and restored disrupted fecal microbiota composition while avoiding bacteremia. This resulted in the safe and effective alleviation of FOLFOX-induced intestinal mucositis in tumor-bearing mice.^31^ In another study, FMT markedly reversed the disruption of gut microbiota caused by ampicillin and/or 5-FU in mice.^225^

In contrast to the results observed for 5-FU, FMT did not exert the expected effects during MTX treatment. An exploratory randomized placebo-controlled trial revealed that the treatment failure rate of FMT in patients with active peripheral psoriatic arthritis receiving MTX was substantially higher than that in the placebo control group.^226^

### 5.4. Others

Exogenous folate supplementation enhances the therapeutic efficacy and reduces the toxicity of MTX. Notably, the gut microbiota of patients with RA exhibit overexpression of metabolic pathways involved in folate biosynthesis. The MTX-induced inhibition of folates has been proposed to reverse this microbial overproduction, potentially increasing microbiome *α*-diversity, which could benefit the gut microbiota of patients with RA.^40^^,^^81^ However, these beneficial effects on the gut microbiota tend to diminish with prolonged folate deprivation, necessitating folate supplementation in cases of MTX overdose.^81^ Given that the toxicity of MTX is linked to its antifolate properties,^227^ folate or its derivatives may be used to prevent the toxic side effects and MTX retention.^46^^,^^72^ In addition to counteracting MTX-mediated inhibition of DHFR, folate mitigates MTX side effects by modulating the gut microbiota.^46^ Specifically, the folate derivative leucovorin increases the abundance of *Bifidobacterium*, which strengthens the intestinal mucosal barrier and exerts anti-inflammatory effects, thereby alleviating MTX-induced gastrointestinal toxicity.^46^

Magnesium isoglycyrrhizinate treatment markedly alleviates MTX-induced intestinal and liver damage; as such, it is routinely used as a hepatoprotective agent in clinical settings to treat DILI.^228^ It alters the composition of the gut microbiota by increasing the abundance of the beneficial bacterium, *Lactobacillus* and decreasing *Muribaculaceae*. This modulation helps to remodel the intestinal barrier and inhibits bacterial translocation to the liver, ultimately reducing MTX-related DILI.^50^

## 6. Future directions

Current research on the complex interactions between antimetabolite drugs and the microbiome has some limitations. A primary challenge is the predominant reliance on cell and animal models with a scarcity of high-quality clinical cohort studies, which limits the strength of evidence. Furthermore, most studies utilize fecal microbiota as a proxy for the entire gut microbiome, despite the limited understanding of compositional variations across different gut regions. The prevalent use of 16S rRNA gene sequencing restricts the depth of the information obtained. Additionally, many studies lack dynamic microbiome profiling during the experimental processes, often comparing only pre- and post-intervention states. The variability in animal models, drug dosages, and dosing intervals further complicates cross-study comparisons. Finally, research on non-bacterial components of the microbiota, such as fungi and viruses, remains insufficient. Future investigations, considering the heterogeneity of patient populations and the increasing use of multidrug regimens, should employ advanced sequencing techniques, such as metagenomics, and incorporate dynamic monitoring of detailed alterations in both bacterial and non-bacterial microbes to yield more comprehensive and precise insights.

## 7. Conclusions

The bidirectional interactions between antimetabolite drugs and the gut microbiota are central to optimizing clinical outcomes. Future research should prioritize leveraging advanced tools including metagenomics and real-time microbial community monitoring to fully elucidate the underlying mechanisms of these interactions. These insights will enable the development of reliable microbial biomarkers for treatment response prediction and innovative microbiota-manipulation strategies. Additionally, future studies must account for patient diversity and combination therapies to translate these insights into precise, clinically applicable antimetabolite regimens.

## Supplementary Material

Certificate_of_editing_FRTJD_1_2_4wqhms6lbt.pdf Certificate_of_editing_FRTJD_1_2_4wqhms6lbt.pdf

Supplementary_tables clean.docx Supplementary_tables clean.docx

## Data Availability

Not applicable.

## Abbreviations

5-FU: 5-fluorouracil
6-MP: mercaptopurine
6-TIMP: 6-thioinosine-monophosphate
6-TX: 6-thioxanthine
ABC: ATP-binding cassette transporter
ADP-H: ADP-heptose
AhR: aryl hydrocarbon receptor
Akt: protein kinase B
AOS: alginate oligosaccharide
AZA: azathioprine
carA/B: carbamoyl phosphate synthetase
CDD: cytidine deaminase
CHP: oyster polysaccharide
CPDG2: carboxypeptidase glutamate 2
CRC: colorectal cancer
CTS: Cryptotanshinone
DAMPA: 2, 4-diamino-*N*-10-methylpteroic acid
DCA: deoxycholic acid
DHFR: dihydrofolate reductase
DHFU: dihydrofluorouracil
DHTS: Dihydrotanshinone
DILI: Drug-Induced Liver Injury
DNA: deoxyribonucleic acid
DR: Dietary restriction
DPYD: dihydropyrimidine dehydrogenase
F/B: Firmicutes/Bacteroidetes
FOX: forkhead box
FOXM1: forkhead box M1
FOXO3: forkhead box O3
FPGS: folylpolyglutamate synthase
FMT: Fecal microbiota transplantation
FOS: fructo-oligosaccharide
FUDR: 5-fluoro-2′-deoxyuridine
FUMP: 5-fluorouridine monophosphate
GLN: glutamine
Glu: glutamic acid
GR: good response
GST: glutathione S-transferase
HPRT: hypoxanthine phosphoribosyltransferase
IAA: indole-3-acetic acid
IBD: inflammatory bowel disease
IFN: Interferon
IL: Interleukin
IPA: indole propionic acid
KEGG: Kyoto Encyclopedia of Genes and Genomes
LPS: lipopolysaccharides
MAPK: mitogen-activated protein kinase
METTL3: methyltransferase-like 3
MTX: methotrexate
MyD88: Myeloid Differentiation Primary Response Gene 88
NAD: nicotinamide adenine dinucleotide
NF-κB: nuclear factor-κB
NGS: next-generation sequencing
NLR: NOD-like receptors
NLRP-3: NOD-like receptor thermal protein domain associated protein 3
NOD: Nucleotide Binding Oligomerization Domain
PDAC: pancreatic ductal adenocarcinoma
PI3K: phosphatidylinositol 3-kinase
PR: poor response
PRR: pattern recognition receptor
RA: rheumatoid arthritis
RFT: reduced folate transporter
RIP2: receptor-interacting protein 2
RNA: ribonucleic acid
SAM: S-adenosylmethionine
SCFA: short-chain fatty acids
SCXN: xylan-stearic acid conjugate
sd-XDH: selenium-dependent xanthine dehydrogenase
SK: skatole
SMCT1: sodium-coupled monocarboxylate transporter 1
STAT3: signal transducer and activator of transcription 3
TG: tioguanine
TGN: thioguanine nucleotides
Th17: T helper cell 17
TIMP: thioinosine monophosphate
TLR: Toll-like receptors
TNF: tumor necrosis factor
Treg: regulatory T cell
tRNA: transfer RNA
TRP: tryptophan
TS: thymidylate synthase
Ub: ubiquitin
UMP: uridine monophosphate
UPP: uracil phosphoribosyltransferase
UroA: urolithin A
UroB: urolithin B
