# Supplementary material for: Exploring the gut microbiome and metabolomic interactions of antimetabolite drugs to optimize therapy
Source: Gut Microbes. 2026 Feb 27;18(1):2638009. doi: 10.1080/19490976.2026.2638009 (PMC12959226; doi:10.1080/19490976.2026.2638009)
Supplement: Certificate_of_editing_FRTJD_1_2_4wqhms6lbt.pdf [file KGMI_A_2638009_SM8391.pdf]

CERTIFICATE OF ENGLISH EDITING

This document certifies that the paper listed below has been edited to ensure that the language is clear and free of errors. The logical presentation of ideas and the structure of the paper were also checked during the editing process. The edit was performed by professional editors at Editage, a division of Cactus Communications, in cooperation with Taylor & Francis Group. The intent of the author's message was not altered in any way during the editing process. The quality of the edit has been guaranteed, with the assumption that our suggested changes have been accepted and have not been further altered without the knowledge of our editors.

Title

Exploring the Gut Microbiome and Metabolomic Interactions of Antimetabolite Drugs to Optimize Therapy

Authors

Jingyang Chen, Yanan Wang, Lei Xu, Xiaona Li*, Libo Zhao*

Order No.

FRTJD_1_2

EDITINGSERVICES
Supporting Taylor & Francis authors

Signature

Prabh Grewal,
Senior Vice President,
Editage

Date of Issue
November 13, 2025
