# Supplementary material for: Exploring the gut microbiome and metabolomic interactions of antimetabolite drugs to optimize therapy
Source: Gut Microbes. 2026 Feb 27;18(1):2638009. doi: 10.1080/19490976.2026.2638009 (PMC12959226; doi:10.1080/19490976.2026.2638009)
Supplement: Supplementary_tables clean.docx [file KGMI_A_2638009_SM8612.docx]

**Table S1** Methods to improve antimetabolite drugs response through gut microbiota

| Intervention | Chemotherapy | Model | Efficacy/Toxicity | Microbiota composition alteration | Mechanism | Reference |
| --- | --- | --- | --- | --- | --- | --- |
| **Prebiotics** |  |  |  |  |  |  |
| Astragalus polysaccharides | 5-FU | BALB/c mice | -/↓ | *Firmicutes, Bacteroidota, Actinobacteriota, Desulfobacterota, Lachnospiraceae_NK4A136_group, Lachnospiraceae_UCG-006, Lachnoclostridium, Roseburia, Blautia, and Odoribacter↑*  *Proteobacteria, Escherichia-Shigella, and Enterococcus↓* | Regulating the composition of the gut microbiota, restoring PUFA metabolism, improving the side effects caused by chemotherapy. | (15) |
| Poria cocos polysaccharides | 5-FU | Apc^Min/+^ mice | -/↓ | *Bacteroides acidifaciens, Bacteroides intestinihominis, Butyricicoccus pullicaecorum, and the genera Lactobacillus, Bifidobacterium, Eubacterium↑*  *Alistipes finegoldii, Alistipes massiliensis, and Alistipes putredinis ↓* | Modulating intestinal inflammation, improving the gut epithelial barrier, and modulating the gut microbiota. | (12) |
| Oyster polysaccharides | 5-FU | KM mice with S180 cells | ↑/↓ | *Verrucomicrobia, Akkermansia, and Odoribacter↑*  *Bacteroidetes, Bacteroides, Prevotellaceae_UCG-001 and Rikenellaceae_RC9_gut_group↓* | Protecting the integrity of the intestinal mucosal barrier, reducing the release of inflammatory factors. | (25) |

**Table S1** (continued)

| Intervention | Chemotherapy | Model | Efficacy/Toxicity | Microbiota composition alteration | Mechanism | Reference |
| --- | --- | --- | --- | --- | --- | --- |
| Albuca Bracteate Polysaccharides | 5-FU | CT26 cells in vitro and BALB/c mice with CT26 cells | ↑/- | *Ruminococcus, Anaerostipes, Oscillospira, Alistipes, and Roseburia↑*  *g_Staphylococcus, s_Staphylococcus sciuri, s_-Ruminoccocus gnavus↓* | Promoting the downregulation of β-Catenin, improving the composition of the gut microbiota, and increasing the levels of fecal SCFAs. | (229) |
| Alginate oligosaccharide | 5-FU | Balb/c mice | -/↓ | *Lachnoclostridium, Muribaculaceae, Oscillospira, Akkermansiaeeae, Bacteroidaceae, and Verrucomicrobiota↑* | Restoring intestinal barrier integrity through enhancing expression of tight junction proteins via MLCK signaling pathway, alleviating intestinal mucosal damage by inhibiting TLR4/MyD88/NF-κB signaling pathway, downregulating the pro-apoptotic protein Bax, upregulating the anti-apoptotic protein Bcl-2, enriching intestinal *Akkermansiaceae*. | (71) |

**Table S1** (continued)

| Intervention | Chemotherapy | Model | Efficacy/Toxicity | Microbiota composition alteration | Mechanism | Reference |
| --- | --- | --- | --- | --- | --- | --- |
| Fructo-oligosaccharides and Arginine | 5-FU | BALB/c mice | -/↓ | *Bacteroides, Anaerostipes, and Lactobacillus↑* | Increasing the number of goblet cells, collagen area, and GPR41 and GPR43 gene expression, modulating intestinal microbiota | (167) |
| Fructo-oligosaccharides | 5-FU | BALB/c mice | -/↓ | NA | Maintaining tight junction expression, reducing inflammatory infiltration and histological scores, and improving SCFA production. | (166) |
| Codonopsis pilosula-derived CP-A (an inulin-type fructan) | 5-FU | Vitro experiments and SD rat models | -/↓ | *Firmicutes, Bacteroidetes, and Lactobacillus↑*  *Proteobacteria, Actinobacteria, and Enterobacteriaceae↓* | Inhibiting the ERK/MLCK/MLC2 pathway, reducing the expression of inflammatory factors, improving the intestinal mucosal barrier, and regulating the intestinal microbial community. | (215) |

**Table S1** (continued)

| Intervention | Chemotherapy | Model | Efficacy/Toxicity | Microbiota composition alteration | Mechanism | Reference |
| --- | --- | --- | --- | --- | --- | --- |
| Berberine | 5-FU | BALB/c mice with CT26 cells | -/↓ | *Akkermansia↑*  *Escherichia/Shigella↓* | Reducing inflammatory factors, inhibiting epithelial cell apoptosis, improving the gut microbiota composition, and upregulating the expression of PI3K/AKT/mTOR. | (13) |
| Berberine | 5-FU | Sprague–Dawley rats | -/↓ | *Firmicutes, unclassified_f_ Porphyromonadaceae, unclassified_f_ Lachnospiraceae, Lactobacillus, unclassified_o_ Clostridiales, Ruminococcus, Prevotella, Clostridium IV↑; Proteobacteria, Escherichia/Shigella↓* | Regulating fecal metabolites to ameliorate 5-Fu induced intestinal mucositis by modifying gut microbiota. | (177) |
| Alpinia katsumadai Hayata Volatile Oil | 5-FU | Kunming mice | -/↓ | *Lactobacillus, Alistipes, Lachnospiraceae_NK4A136_group, Monoglobus, Roseburia, Fusicatenibacter, Butyricicoccus, Phascolarctobacterium, Incertae_Sedis, Bilophila, and CAG-56↑; Erysipelatoclostridium↓* | Regulating the expressions of tight junction proteins via modulation of GC/GR and mPGES-1/PGE2/EP4 pathway. | (16) |

**Table S1** (continued)

| Intervention | Chemotherapy | Model | Efficacy/Toxicity | Microbiota composition alteration | Mechanism | Reference |
| --- | --- | --- | --- | --- | --- | --- |
| Volatile Oil from Amomi Fructus（bornyl acetate） | 5-FU | Spraque–Dawley rats | -/↓ | *Lactobacillus, Bifidobacterium↑*  *Escherichia, Bacteroides, Helicobacter, Desulfovibrio, Ruminococcus, Parabacteroides, and Clostridium↓* | Inhibiting apoptosis and alleviating the endoenteritis by downregulating p38 MAPK and caspase-3 expression, strengthening the intestinal mucosal barrier by increasing zonula occludin-1 and occludin expression, reducing the amount of pathogenic bacteria and increasing the abundance of probiotics. | (219) |
| Babao Dan | 5-FU | BALB/c mice | -/↓ | *Firmicutes, Bacilli, Lactobacillales, Roseburia, unidentified_Corynebacteriaceae, Aerococcus, Blautia, Jeotgalicoccus, Odoribacter, Roseburia, Rikenella, Intestinimonas, unidentified_Lachnospiraceae, Enterorhabdus, Ruminiclostridium↑*  *Bacteroidia, Bacteroidales, Bacteroides↓* | Reducing the concentration of LPS in the fecal suspension and serum, and inhibiting TLR4/MyD88/NF-κB pathway activation, repairing the disruption of the gut microbiota caused in a time-dependent way. | (22) |

**Table S1** (continued)

| Intervention | Chemotherapy | Model | Efficacy/Toxicity | Microbiota composition alteration | Mechanism | Reference |
| --- | --- | --- | --- | --- | --- | --- |
| Wumei pills | 5-FU | BABL/C mice | -/↓ | *Lactobacilli↑*  *Bacteroides, Helicobacter, and Parabacteroides↓* | Reducing the levels of TNF-α, IL-1β, IL-6, and MPO and inhibiting the expression of the TLR4/MyD88/NF-κB pathway proteins, repairing the integrity of the mucosal barrier of mice, regulating the intestinal flora, and increasing the levels of SCFA. | (230) |
| Casuarinin from Melastoma malabathricum | 5-FU | C57BL/6 mice | -/↓ | *Actinobacteria, Lachnospiraceae_NK4A136_group and Lactobacillus murinus↑*  *Candidatus Arthromitus↓* | Modulating inflammation, intestinal barrier dysfunction, and gut microbiota dysbiosis. | (178) |
| Cryptotanshinone | 5-FU | BALB/c mice with AOM+DSS induction | -/↓ | *g*_*Lactobacillus, g_Alistipes, and g*_*Odoribacter↑*  *g_norank, f_Muribaculaceae↓* | Regulating fecal flora associated lipid metabolism. | (172) |

**Table S1** (continued)

| Intervention | Chemotherapy | Model | Efficacy/Toxicity | Microbiota composition alteration | Mechanism | Reference |
| --- | --- | --- | --- | --- | --- | --- |
| Dihydrotanshinone | 5-FU | C57BL/6 mice | -/↓ | *g_Akkermansia↑* | Restoring disordered fecal microbiota community. | (174) |
| Patchouli alcohol | 5-FU | Sprague-Dawley rats | -/↓ | *Bifidobacterial and Lactobacilli↑*  *Bacteroides, Helicobacter, and Parabacteroides↓* | Maintaining intestinal mucosal barrier via suppressing TLR2/MyD88/NF-κB pathway, participating in regulation of the microbiota structure. | (19) |
| Fungal proteins from Hericium erinaceus | 5-FU | Balb/C mice with CT-26 wt cancer cells | ↑/- | *Bifidobacterium, Gemellales, Blautia, Sutterella, Anaerostipes, Roseburia, Lachnobacterium, Lactobacillus, and Desulfovibrio↑; Parabacteroides, Flavobacteriaceae, Christensenellaceae, Anoxybacillus, Aggregatibacter, Comamonadaceae, Planococcaceae, Desulfovibrionaceae, Sporosarcina, Staphylococcus, Aerococcaceae, and Bilophila↓* | Improving the microbiota composition, the immune inflammatory response, and homeostasis. | (231) |

**Table S1** (continued)

| Intervention | Chemotherapy | Model | Efficacy/Toxicity | Microbiota composition alteration | Mechanism | Reference |
| --- | --- | --- | --- | --- | --- | --- |
| Bupi Hewei decoction | 5-FU | Sprague-Dawley rats | -/↓ | *Bacteroidetes and Prevotellaceae-Alloprevotella species↑*  *Proteobacteria, Escherichia-Shigella, Ruminococcaceae NK4A214, and Ruminococcaceae UCG-005 species↓* | Improving the intestinal immune balance and reducing intestinal inflammation by targeting T helper cell/T regulatory cell-associated factors. | (232) |
| Diadzein | 5-FU | BLAB/c mice | -/↓ | *Lactobacillus↑*  *Escherichia coli↓* | Inhibition of oxidative stress and inflammatory mediators. | (233) |
| Saikosaponin-A | 5-FU | BALB/c mice | -/↓ | *Lactobacillus spp.↑*  *Escherichia coli↓* | Inhibiting pro-inflammatory mediators (TNF-α, COX-2, IL-1β and IL-6) and apoptotic markers (p-JNK, Casp-3). | (179) |
| Carboxymethyl pachyman | 5-FU | Balb/c mice with CT26 cells | -/↓ | *Bacteroidetes, lactobacilli, and butyric acid-producing and acetic acid-producing bacteria↑* | Regulation of the NF-κB, Nrf2-ARE and MAPK/P38 pathways. | (217) |

**Table S1** (continued)

| Intervention | Chemotherapy | Model | Efficacy/Toxicity | Microbiota composition alteration | Mechanism | Reference |
| --- | --- | --- | --- | --- | --- | --- |
| L-Glutamine | 5-FU | Institute of Cancer Research (ICR) mice | -/↓ | *Firmicutes, Oscillibacter valericigenes, Kineothrix alysoides, and Ligilactobacillus murinus↑*  *Bacteroidetes, Verrucomicrobia, E. coli, B. caccae, and A. muciniphila↓* | Inhibiting the Toll-like receptor 4/nuclear factor kappa B (TLR4/NF-κB) pathway, modulating nuclear factor erythroid 2-related factor 2/heme oxygenase 1 (Nrf2/HO-1) oxidative stress proteins, and increasing mammalian target of rapamycin (mTOR) levels. | (14) |
| Ursolic acid | Gemcitabine | BALB/c nude mice with pancreatic cancer cells | ↑/- | *Erysipelatoclostridium↑*  *Ruminiclostridium, Mucispirillum↓* | Suppressing the RAGE/NF-κB/MDR1 cascade and consequently inhibiting subcutaneous tumor growth. | (234) |
| Gum odina -sodium alginate conjugate | Capecitabine | Colon cancer on Swiss albino mice | ↑/- | NA | Raising intra-tumoral capecitabine concentration, slowing drug elimination, improving anti-tumor immunity. | (235) |

**Table S1** (continued)

| Intervention | Chemotherapy | Model | Efficacy/Toxicity | Microbiota composition alteration | Mechanism | Reference |
| --- | --- | --- | --- | --- | --- | --- |
| Xylan-stearic acid conjugate | Capecitabine | Balb/c mice with CT26 cells | ↑/- | *Clostridia, Lachnospiraceae, Ruminococccaceae, Roseburia, Bifidobacterium, Akkermansia and Faecalibaculum↑*  *Desulfovibro↓* | Increasing the accumulation of drugs within tumors, promoting the maturation of dendritic cells, reducing Tregs in the tumor microenvironment, and increasing CD8+ T cells | (170) |
| Prebiotic fibre mixtures | 5-FU | vitro model of the colon | ↑/↓ | *Bifidobacterium, Lactobacillus, Anaerostipes, Weissella, Olsenella, Senegalimassilia↑; Klebsiella, Enterobacter↓* | Regulating the gut microbiota dysbiosis induced by 5-FU. | (236) |
| **Probiotics** |  |  |  |  |  |  |
| Probiotic supplementation | 5-FU | C57BL/6 J mice with DMH induction | -/↓ | NA | Increasing the production of Ki-67 cell-proliferation marker, modulating chronic inflammatory process by inhibiting NF-κB expression and mitigating mucin depletion. | (186) |

**Table S1** (continued)

| Intervention | Chemotherapy | Model | Efficacy/Toxicity | Microbiota composition alteration | Mechanism | Reference |
| --- | --- | --- | --- | --- | --- | --- |
| Lactobacillus casei variety rhamnosus and Lactobacillus reuteri DSM 17938 | 5-FU | BALB/c mice | -/↓ | *Proteobacteria↑*  *Bacteroidetes↓* | Modulating the abundance and diversity of gut microbiota. | (181) |
| Pediococcus pentosaceus PP34 | 5-FU | C57BL/6 mice | -/↓ | *Defluviitaleaceae_UCG-011, Family_XIII_AD3011_group, Pediococcus, Ruminiclostridium_1, Fusobacterium, Akkermansia, and Parasutterella↑* | Inhibiting oxidative stress and restoring the gut microbiota. | (182) |
| Akkermansia muciniphila and its outer membrane protein Amuc_1100 | 5-FU | C57BL/6 mice | -/↓ | *Verrucomicrobia, A. muciniphila, and Blautia↑*  *Eubacterium coprostanoligene↓* | Reducing intestinal mucositis, restoring intestinal epithelial integrity, affecting the mRNA levels of inflammatory cytokines, affecting the activation of NLRP-3 inflammatory vesicles, regulating the composition of the intestinal microbiota. | (133) |

**Table S1** (continued)

| Intervention | Chemotherapy | Model | Efficacy/Toxicity | Microbiota composition alteration | Mechanism | Reference |
| --- | --- | --- | --- | --- | --- | --- |
| Lactobacillus casei Variety rhamnosus Probiotic | FOLFOX | BALB/c mice with CT26 cells | -/↓ | *Bacteroidetes↑*  *Firmicutes↓* | Modulation of gut microbiota and proinflammatory responses with suppression of intrinsic apoptosis in intestinal injury. | (218) |
| Dairy bacterium Lactococcus lactis and recombinant strain secreting human antimicrobial PAP | 5-FU | BALB/c mice | -/↓ | *Akkermansia, Lactobacillales, Peptococcaceae, and RF39↑*  *Actinobacteria, Clostridiaceae, Enterobacteriaceae and Corynebacterium↓* | Regulating the gut microbiota dysbiosis. | (237) |
| Bifidobacterium bifidum G9-1 | 5-FU | ICR (CD1) mice | -/↓ | *Firmicutes↑*  *Bacteroidetes↓* | Attenuation of inflammatory responses via improve dysbiosis. | (23) |
| Saccharomyces boulardii | 5-FU | Swiss mice | -/↓ | NA | Reducing the inflammation and dysfunction of the gastrointestinal tract in intestinal mucositis induced by 5-FU. | (185) |

**Table S1** (continued)

| Intervention | Chemotherapy | Model | Efficacy/Toxicity | Microbiota composition alteration | Mechanism | Reference |
| --- | --- | --- | --- | --- | --- | --- |
| Saccharomyces boulardii(CNCM I-745) | MTX | CIA Sprague-Dawley (SD) rat model | ↑/- | NA | Enhancing bone and joint integrity, modulating gut microbiota, and mitigating proinflammatory cytokine levels, decreasing the permeability of the intestines and promoting the production of gut tight-junction proteins. | (184) |
| Bacteroides fragilis | MTX | CIA gut-deficient mice model | ↑/- | *Bacteroides fragilis↑* | Supplementation of butyrate. | (136) |
| Mucin degrader Akkermansia muciniphila | MTX | C57BL/6 mice | -/↓ | *Bacteroidetes, Proteobacteria, Muribaculum, Alistipes, Akkermansia, Helicobacter, and Desulfovibrio↑*  *Firmicutes↓* | Accelerating the proliferation of Lgr5+ ISCs and promoting the differentiation of Paneth cells and goblet cells in the small intestine. | (183) |

**Table S1** (continued)

| Intervention | Chemotherapy | Model | Efficacy/Toxicity | Microbiota composition alteration | Mechanism | Reference |
| --- | --- | --- | --- | --- | --- | --- |
| Escherichia coli O83 | MTX | Adjuvant induced arthritis Lewis rats | ↑/- | NA | Inhibiting both inflammation and destructive arthritis-associated changes. | (238) |
| Probiotic blend | Gemcitabine | Pancreatic cancer xenografted mice | -/↓ | *Eubacteriaceae, Ruthenibacterium, Faecalicatena, Pseudobutyrivibrio, and Roseburia↑* | Bacteria that produce butyrate and other beneficial short-chain fatty acids increase, amino acids significantly decrease, and serum choline is notably reduced. | (239) |
| Probiotics mixture | Gemcitabine and cisplatin | Urothelial cancer C3H mice | ↑/- | *Lactobacillus and Bifidobacterium↑* | Activating antigen-presenting cells and recruiting cytotoxic T cells, thereby enhancing anti-tumor effects. | (187) |
| Probiotics mixture | Gemcitabine | Pancreatic cancer mice | ↑/- | NA | Decreasing aspartate aminotransferase and alanine aminotransferase levels. | (240) |

**Table S1** (continued)

| Intervention | Chemotherapy | Model | Efficacy/Toxicity | Microbiota composition alteration | Mechanism | Reference |
| --- | --- | --- | --- | --- | --- | --- |
| Lactobacillus rhamnosus R0011 | capecitabine | Colon Cancer in Male Balb/c Mice | ↑/- | NA | Enhancing Bax/Bcl-2 ratio and caspase-3 level. | (241) |
| **Synbiotics** |  |  |  |  |  |  |
| Resistant Starch-Encapsulated Probiotics | 5-FU | BALB/cByJNarl mice with CT26 cells | ↑/↓ | *Firmicutes, Bacilli, Verrucomicrobiae, Desulfovibrionia, Oscillospiraceae uncultured and Mucispirillum↑*  *Bacteroidetes, Coriobacteriia, Saccharimonadia, Bacteroidia, Coriobacteriales, Saccharimonadles, Bacteroidales, Enterorhabdus, RF39, Erysipelotrichaceae, and Candidatus Saccharimonas↓* | Modulating tumor-derived proinflammatory cytokines and regulating the NF-κB pathway within the tumor tissue, modulating the structure and diversity of the gut microbiota. | (191) |
| Probiotics mixture（Probiotics + inulin/lactoferrin） | gemcitabine+ nab-paclitaxel | Pancreatic cancer mice | -/↓ | *Actinobacteria, Propionibacteriaceae, Bacteroidaceae and Lactobacillaceae↑* | Restoring a favorable microbiota composition. | (192) |

**Table S1** (continued)

| Intervention | Chemotherapy | Model | Efficacy/Toxicity | Microbiota composition alteration | Mechanism | Reference |
| --- | --- | --- | --- | --- | --- | --- |
| **Postbiotics** |  |  |  |  |  |  |
| Paraprobiotic Lacticaseibacillus rhamnosus | 5-FU | BALB/c mice | -/↓ | NA | Preserving the villi and intestinal crypts, reducing the inflammatory infiltrate, and increasing the mucus secretion, Muc2 gene expression, and Treg cells frequency. | (196) |
| Lactobacillus rhamnosus GG cell-free supernatant | 5-FU | Human colon and melanoma cancer cell lines | ↑/- | NA | Containing one or more bioactive molecules with anti-cancer activity which sensitize cancer cells to chemotherapeutic drugs. | (195) |
| Urolithin B | capecitabine | AOM/DSS induced C57BL/6 mice | ↑/- | *Verrucomicrobiota, Desulfobacterota, Akkermansiaceae↑*  *Bacteroides, Alloprevotella↓* | Improving the colorectal intestinal hematochezia by shaping gut microbiota. | (197) |

**Table S1** (continued)

| Intervention | Chemotherapy | Model | Efficacy/Toxicity | Microbiota composition alteration | Mechanism | Reference |
| --- | --- | --- | --- | --- | --- | --- |
| **Dietary interventions** |  |  |  |  |  |  |
| Dietary restriction | 5-FU | C57BL/6j mice | -/↓ | *Lactobacillales, Lactobacillaceae and Lactobacillus↑* | Protecting against the loss of lysozyme and increasing the content of *Lactobacillus*, resulting in a significant inhibition of intestinal opportunistic pathogens and their translocation. | (199) |
| Fibre-rich diet | 5-FU | C57Bl/6 mice | -/↓ | *Bacteroidaceae and Akkermansiaceae↑* | Altering the composition of the gut microbiota, reducing the expression of GFAP in the CA1 region of the hippocampus and the midbrain. | (202) |
| Whey-based diet containing medium chain triglycerides | MTX | Wistar rats and DAMA model | ↑/↓ | *Peptostreptococcaeceae, Muribaculaceae↑*  *Ruminococcaceae↓* | Modulating the gut microbiota and protecting the intestinal mucosa. | (203) |

**Table S1** (continued)

| Intervention | Chemotherapy | Model | Efficacy/Toxicity | Microbiota composition alteration | Mechanism | Reference |
| --- | --- | --- | --- | --- | --- | --- |
| Dietary restriction | MTX | C57BL/6J mice | -/↓ | *Lactobacillus genus↑* | Reducing intestinal inflammation, preserving the number of basal crypt PCNA-positive cells, and protecting the function of intestinal stem cells. | (200) |
| Ketogenic diet | gemcitabine | Pancreatic tumor-bearing KPC mice | -/↓ | *Firmicutes, Erysipelatoclostridium↑* | Inhibition of ERK and AKT pathways, regulation of fatty acid metabolism and the modulation of the gut microbiota. | (242) |
| **FMT** |  |  |  |  |  |  |
| Fecal microbiota transplantation | FOLFOX | BALB/c mice with CT26 cells | -/↓ | *Bacteroidetes↑*  *Firmicutes↓* | Regulating the gut microbiota TLR-MyD88-NF-κB signaling pathway. | (208) |
| Fecal microbiota transplantation | 5-FU | C57BL/6J mice | -/↓ | NA | Reversing disruption of the intestinal microbiota. | (209) |

**Table S1** (continued)

| Intervention | Chemotherapy | Model | Efficacy/Toxicity | Microbiota composition alteration | Mechanism | Reference |
| --- | --- | --- | --- | --- | --- | --- |
| **Others** |  |  |  |  |  |  |
| Mild moxibustion | 5-FU | Sprague-Dawley rats | -/↓ | NA | Ameliorating mucosal damage and reducing inflammation. | (132) |
| Neutrophil elastase inhibitor (MPH-966) | 5-FU | C57BL/6 mice | -/↓ | *Muribaculaceae, Ruminococcaceae, and Eggerthellaceae↑*  *Candidatus arthromitus↓* | Regulating abnormal inflammatory responses, intestinal barrier dysfunction, and gut microbiota imbalance. | (243) |
| Leucovorin | MTX | Balb/c mice | -/↓ | *Bifidobacterium↑* | Alleviating gut microbiota imbalance and increasing the abundance of *Bifidobacterium*. | (212) |
| Vitamins C and B2 | MTX | Wistar rats | -/↓ | *Blautia coccoides and Roseburia intestinalis↑* | Enhancing anaerobic bacterial growth in vitro. | (244) |
| Magnesium isoglycyrrhizinate | MTX | C57BL/6 mice | -/↓ | *Lactobacillus↑*  *Muribaculaceae↓* | Altering the gut microbiota composition and inhibiting bacterial translocation to the liver. | (214) |

**Abbreviations:** 5-FU: 5-fluorouracil; AKT: protein kinase B; AOM: Azoxymethane; Bax: Bcl-2-associated X protein; Bcl-2: B-cell lymphoma 2; CA1: cornu Ammonis 1; Casp-3: Caspase-3; CIA: collagen-induced arthritis; COX-2: cyclooxygenase-2; DSS: dextran sodium sulfate; EP4: prostaglandin E receptor 4; ERK: extracellular signal-regulated kinase; FMT: fecal microbiota transplantation; GFAP: glial fibrillary acidic protein; GC: glucocorticoid; GR: glucocorticoid receptor; GPR: G protein-coupled receptor; HO-1: heme oxygenase-1; ICR: Institute of Cancer Research; IL-1β: interleukin-1β; IL-6: interleukin-6; ISC: intestinal stem cell; JNK: c-Jun N-terminal kinase; KM: Kunming; LPS: Lipopolysaccharide; MAPK: mitogen-activated protein kinase; MLCK: myosin light chain kinase; MLC2: myosin light chain 2; MDR1: multidrug resistance 1; MPH-966: neutrophil elastase inhibitor; MPO: myeloperoxidase; mPGES-1: microsomal prostaglandin E synthase-1; mTOR: mammalian target of rapamycin; MTX: methotrexate; MyD88: myeloid differentiation primary response 88; nab-paclitaxel: nanoparticle albumin-bound paclitaxel; NLRP-3: NOD-like receptor pyrin domain-containing 3; NF-κB: nuclear factor kappa B; Nrf2: nuclear factor erythroid 2-related factor 2; PAP: peptide antimicrobial protein; PCNA: proliferating cell nuclear antigen; PI3K: phosphatidylinositol 3-kinase; PGE2: prostaglandin E2; PUFA: polyunsaturated fatty acid; RAGE: receptor for advanced glycation end products; SCFA: short-chain fatty acid; SD: Sprague-Dawley; TLR: Toll-like receptor; TNF-α: tumor necrosis factor-α; UCG: unclassified genus; ↑: increased levels/index/ratio; ↓: reduced levels/index/ratio; -: no obvious variation;

**Legend:** FOLFOX: leucovorin, oxaliplatin, and 5-fluorouracil; NA: not acquired;

Dotted lines divide the table into sections based on different intervention strategies.

**References:**

229. Yuan X, Xue J, Tan Y, Yang Q, Qin Z, Bao X, Li S, Pan L, Jiang Z, Wang Y, et al. Albuca bracteate polysaccharides synergistically enhance the anti-tumor efficacy of 5-Fluorouracil against colorectal cancer by modulating β-Catenin signaling and intestinal flora. Front Pharmacol. 2021;12:736627. doi: 10.3389/fphar.2021.736627.

230. Lu DX, Liu F, Wu H, Liu HX, Chen BY, Yan J, Sun Z. Wumei pills attenuates 5-fluorouracil-induced intestinal mucositis through toll-like receptor 4/myeloid differentiation factor 88/nuclear factor-κB pathway and microbiota regulation. World J Gastroenterol. 2022;28(32):4574–4599. doi: 10.3748/wjg.v28.i32.4574.

231. Wang D, Zhu X, Tang X, Li H, Yizhen X, Chen D. Auxiliary antitumor effects of fungal proteins from hericium erinaceus by target on the gut microbiota. J Food Sci. 2020;85(6):1872–1890. doi: 10.1111/1750-3841.15134.

232. Sun Z, Hu Y, Wang Y, Feng J, Dou Y. Bupi hewei decoction ameliorates 5-fluorouracil-induced intestinal dysbiosis in rats through T helper 17/T regulatory cell signaling pathway. J Tradit Chin Med. 2020;40(1):38–48.

233. Atiq A, Shal B, Naveed M, Khan A, Ali J, Zeeshan S, Al-Sharari SD, Kim YS. Diadzein ameliorates 5-fluorouracil-induced intestinal mucositis by suppressing oxidative stress and inflammatory mediators in rodents. Eur J Pharmacol. 2019;843:292–306. doi: 10.1016/j.ejphar.2018.12.014.

234. Li ZY, Chen SY, Weng MH, Yen GC. Ursolic acid restores sensitivity to gemcitabine through the RAGE/NF-κB/MDR1 axis in pancreatic cancer cells and in a mouse xenograft model. J Food Drug Anal. 2021;29(2):262–274.

235. Hazra A, Tudu M, Mohanta A, Samanta A. Gum odina prebiotic induced gut modulation for the treatment of colon cancer on Swiss albino mice: by using capecitabine loaded biopolymeric microsphere. Int J Biol Macromol. 2024;267(Pt 2):131410. doi: 10.1016/j.ijbiomac.2024.131410.

236. Ziemons J, Hillege LE, Aarnoutse R, de Vos-Geelen J, Valkenburg-van Iersel L, Mastenbroek J, van Geel R, Barnett DJM, Rensen SS, van Helvoort A, et al. Prebiotic fibre mixtures counteract the manifestation of gut microbial dysbiosis induced by the chemotherapeutic 5-Fluorouracil (5-FU) in a validated in vitro model of the colon. BMC Microbiol. 2024;24(1):222. doi: 10.1186/s12866-024-03384-4.

237. Carvalho R, Vaz A, Pereira FL, Dorella F, Aguiar E, Chatel JM, Bermudez L, Langella P, Fernandes G, Figueiredo H, et al. Gut microbiome modulation during treatment of mucositis with the dairy bacterium lactococcus lactis and recombinant strain secreting human antimicrobial PAP. Sci Rep. 2018;8(1):15072. doi: 10.1038/s41598-018-33469-w.

238. Rovenský J, Stancíková M, Svík K, Utesený J, Bauerová K, Jurcovicová J. Treatment of adjuvant-induced arthritis with the combination of methotrexate and probiotic bacteria escherichia coli O83 (Colinfant). Folia Microbiol (Praha). 2009;54(4):359–363. doi: 10.1007/s12223-009-0045-2.

239. Panebianco C, Pisati F, Ulaszewska M, Andolfo A, Villani A, Federici F, Laura M, Rizzi E, Potenza A, Latiano TP, et al. Tuning gut microbiota through a probiotic blend in gemcitabine-treated pancreatic cancer xenografted mice. Clin Transl Med. 2021;11(11):e580. doi: 10.1002/ctm2.580.

240. Chen SM, Chieng WW, Huang SW, Hsu LJ, Jan MS. The synergistic tumor growth-inhibitory effect of probiotic lactobacillus on transgenic mouse model of pancreatic cancer treated with gemcitabine. Sci Rep. 2020;10(1):20319. doi: 10.1038/s41598-020-77322-5.

241. Rahimpour M, Ashabi G, Rahimi AM, Halimi S, Panahi M, Alemrajabi M, Nabavizadeh F. Lactobacillus rhamnosus R0011 treatment enhanced efficacy of capecitabine against colon cancer in Male balb/c mice. Nutr Cancer. 2022;74(7):2622–2631. doi: 10.1080/01635581.2021.2014901.

242. Cortez NE, Rodriguez Lanzi C, Hong BV, Xu J, Wang F, Chen S, Ramsey JJ, Pontifex MG, Müller M, Vauzour D, et al. A ketogenic diet in combination with gemcitabine increases survival in pancreatic cancer KPC mice. Cancer Res Commun. 2022;2(9):951–965. doi: 10.1158/2767-9764.CRC-22-0256.

243. Chen KJ, Chen YL, Ueng SH, Hwang TL, Kuo LM, Hsieh PW. Neutrophil elastase inhibitor (MPH-966) improves intestinal mucosal damage and gut microbiota in a mouse model of 5-fluorouracil-induced intestinal mucositis. Biomed Pharmacother. 2021;134:111152. doi: 10.1016/j.biopha.2020.111152.

244. da Silva Ferreira AR, Wardill HR, Havinga R, Tissing WJE, Harmsen HJM. Prophylactic treatment with vitamins C and B2 for methotrexate-induced gastrointestinal mucositis. Biomolecules. 2020;11(1):34. doi: 10.3390/biom11010034.
